# Polyisobutylenes with Controlled Molecular Weight and Chain-End Structure: Synthesis and Actual Applications

**DOI:** 10.3390/polym15163415

**Published:** 2023-08-15

**Authors:** Ilya E. Nifant’ev, Sofia A. Korchagina, Maria S. Chinova, Alexander N. Tavtorkin

**Affiliations:** A.V. Topchiev Institute of Petrochemical Synthesis RAS, 29 Leninsky Pr., 119991 Moscow, Russia; korchagina@ips.ac.ru (S.A.K.); chinova@yandex.ru (M.S.C.); tavtorkin@yandex.ru (A.N.T.)

**Keywords:** cationic polymerization, *exo*-olefinic group, highly reactive polyisobutylene, isobutylene, polymer functionalization, succinic anhydrides, succinimides

## Abstract

The polymerization of isobutylene allows us to obtain a wide spectrum of polyisobutylenes (PIBs) which differ in their molecular weight characteristics and the chemical structure of chain-end groups. The bulk of the PIBs manufactured worldwide are highly reactive polyisobutylenes (HRPIBs) with –C(Me)=CH_2_ end-groups and low-molecular weights (*M*_n_ < 5 kDa). HRPIBs are feedstocks that are in high demand in the manufacturing of additives for fuels and oils, adhesives, detergents, and other fine chemicals. In addition, HRPIBs and CMe_2_Cl-terminated PIBs are intensively studied with the aim of finding biomedical applications and for the purpose of developing new materials. Both chain control (molecular weight and dispersity) and chemoselectivity (formation of *exo*-olefinic or –CMe_2_Cl groups) should be achieved during polymerization. This review highlights the fundamental issues in the mechanisms of isobutylene polymerization and PIB analysis, examines actual catalytic approaches to PIBs, and describes recent studies on the functionalization and applications of HRPIBs and halogen-terminated PIBs.

## 1. Introduction

The polymerization of isobutylene (IB) has been used in the production of high- (*M*_n_ > 100 kDa) and medium- (*M*_n_ = 40–100 kDa) molecular weight (MW) polyisobutylenes (PIBs) and IB/isoprene copolymers (butyl rubber) [1,2,3]. Due to their remarkable impermeable property, PIBs are extensively used in many applications, such as the inner tubes and layers of tires [4]. However, the low-MW PIB (*M*_n_ < 5 kDa), possessing the *exo*-olefinic end-group –C(Me)=CH_2_, holds the major share in the PIB market (more than 70% [2]). The PIB market was valued at USD 2.87 billion in 2021 [5] and is projected to reach USD 3.89 billion by 2028; it is expected to grow at an annual rate of 4.4% from 2021 to 2028. BASF, Braskem S.A., Chevron, Daelim Industrial Petrochemical Division, INEOS, Infineum, Kemat Polybutenes, Kothari Petrochemicals, Lubrizol Corp., SIBUR Holding, and TPC Group are the key players in the global PIB market [4]. Recently, in response to the rising global demand for high-quality medium-molecular weight PIBs, the leading chemical companies have increased their production capacity for these polymers [6,7].

**Scheme 1:**
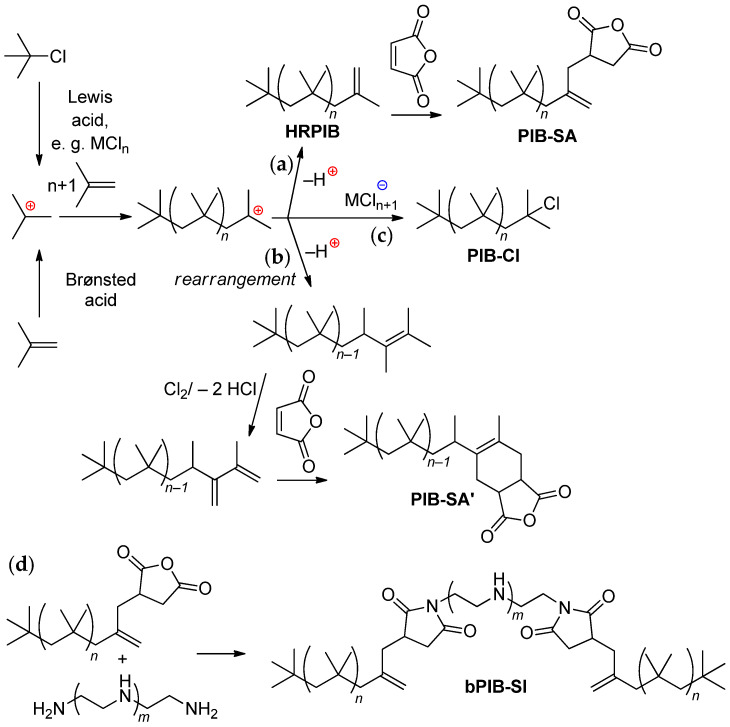
Polymerization of isobutylene with a formation of HRPIB and PIB-SA (**a**); PIB with internal olefinic group and PIB-SA′ (**b**); and PIB-Cl (**c**). Synthesis of bPIB-SI (**d**).

Low-MW PIBs have been widely used in the production of different fine chemicals, the bulk of which are ashless dispersants for motor oils and fuels. PIB-based dispersants represent bis-PIB succinimides (bPIB-SI); their synthesis includes two stages (Scheme 1), viz. the Alder-ene reaction between PIB and maleic anhydride (MA) with the formation of polyisobutylene-succinic anhydrides (PIB-SAs), followed by the reaction of the PIB-SAs with H_2_N(CH_2_CH_2_NH)*_n_*CH_2_CH_2_NH_2_ (*n* = 1–4) and other amines [8,9,10]. The rate of the Alder-ene reaction depends significantly on the structure of the chain-end unsaturated fragment; the *exo*-olefinic group is the most reactive, and low-MW PIB containing >80% methylene fragments are known as highly reactive polyisobutylenes (HRPIBs) (Scheme 1a). In addition to manufacturing ashless dispersants [11], PIB-SAs have found application in the production of antifouling agents, detergents, and corrosion inhibitors [12]. The synthesis of HRPIBs has been successfully commercialized; they are produced as ULTRAVIS (BP Chemicals, London, UK), GLISSOPAL (BASF) RB HR PIB (RB Products, Inc., Houston, TX, USA), and others, with the use of BF_3_/ROH or BF_3_/H_2_O initiators [13,14,15].

It is important to note that the synthesis of PIB-SA was previously based on the use of low-MW polyisobutylenes containing internal C=C fragments, obtained in the presence of AlCl_3_ or chloroalkylaluminum initiators. This approach included an additional stage of chlorination/dehydrochlorination, followed by a Diels–Alder reaction of the diene intermediate with MA (Scheme 1b) [16,17,18]. This method currently seems less applicable due to ecological requirements; however, the same requirements necessitate the search for an alternative for toxic and corrosive BF_3_ [19] in the production of HRPIB.

It is not necessary to dismiss the low-temperature controlled ‘living’ polymerization of IB initiated by *tert*-alkyl chlorides (PhCMe_2_Cl and, later, *^t^*BuCH_2_CMe_2_Cl) with a formation of CMe_2_Cl-terminated PIBs [20] (Scheme 1c).

**Figure 1:**
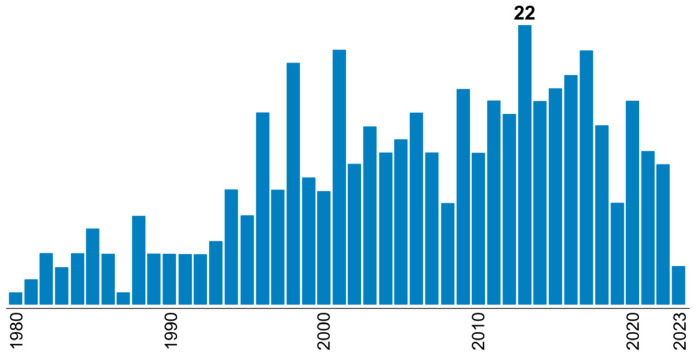
The results of the reference search for “Reactive” AND “Polyisobutylene” (SciFinder, 15 June 2023).

According to the statistical data presented in a recent bibliographic review [21], the topic of IB polymerization is still relevant, and the focus is on the search for available, convenient, and “green” catalysts to produce HRPIB and reactive PIBs, and their applications (Figure 1). The cationic polymerization of IB at ambient temperature was reviewed by Kostjuk et al. in 2013 [22]. In a more recent work [23], the chain transfer catalytic polymerization of IB with a formation of HRPIB had been discussed. From the 1990s to the early 2000s, studies of catalysts and initiators involving weakly coordinating anions, such as perfluoroaryl borates and similar systems, attracted researchers’ attention [15,24]; however, these approaches were not particularly effective in the synthesis of relatively low-MW HRPIB [25]. Nevertheless, the use of cationic initiators in combination with weakly coordinating anions remains present in the synthesis of high-MW PIBs with *exo*-olefinic end-groups [26,27,28]. PIB-Cl can be transformed into HRPIBs via quenching with bases [29,30], but this method seems overly complicated and costly in comparison with BF_3_-based approaches applied to low-MW HRPIBs.

In a recent review [2], Kostjuk et al. have suggested that newly developed IB polymerization catalysts may be divided into three generations. The first-generation catalysts are complexes of metal halides with ethers; these complexes are poorly soluble in hydrocarbons. The latter disadvantage was overcome by the development of second-generation catalysts, complexes of alkylaluminum dichlorides with ethers. The third-generation catalysts are heterogeneous systems. In the present paper, we summarize and discuss the fundamental issues of the mechanisms of IB polymerization and PIB characterization, and analyze actual catalytic approaches to HRPIB in comparison with industrial catalysts. Our review is based on articles and patents published since 2010, and particular attention is given to recent articles not included in previous reviews.

However, the negative trend visible in Figure 1 has nothing in common with the applications of HRPIBs and PIB-Cl. Recent studies of HRPIB and PIB-Cl functionalization, as well as applications of the materials obtained, are also the subject of our review.

## 2. Analysis of PIBs and Mechanism of IB Polymerization

### 2.1. Analysis of PIBs

The microstructure of the products of IB polymerization is an objective basis for the development of catalysts providing desired molecular weight characteristics and a sufficiently high content of *exo*-olefinic groups. The *M*_n_ requirements of HRPIB (several kDa) greatly simplify the chain-end analysis of the polymers using NMR spectroscopy; however, the possibility of chain scission processes occurring during IB polymerization requires the use of mass spectrometry for the analysis of PIB. The basic methods of the study of PIBs have been developed long ago, but we thought it appropriate to include the results of the earlier studies in our review with recent add-ons.

#### 2.1.1. NMR Spectroscopy

**Figure 2:**
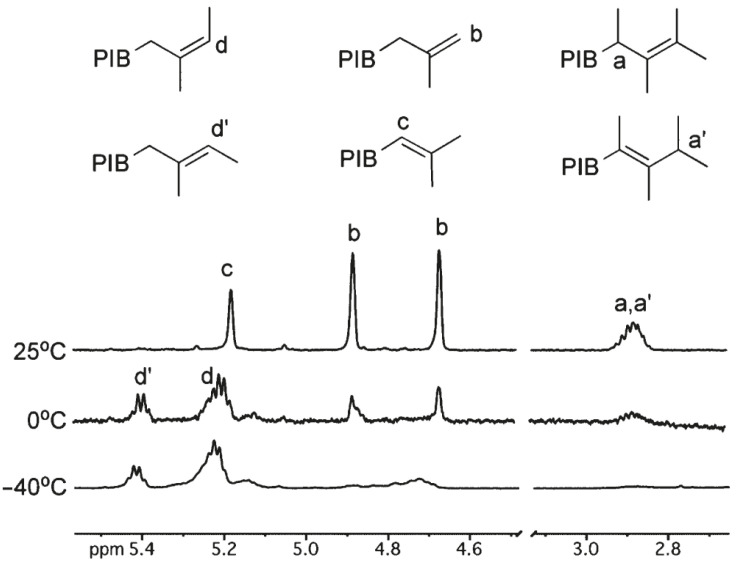
Effect of temperature on olefin end-group distribution during ionization of PIB-Cl with EtAlCl_2_ in hexanes. [PIB-Cl] = 0.015 M, [EtAlCl_2_] = 0.015 M, and [DTBP] = 0.015 M. The letters a, a′ indicate characteristic signals of >CH– fragments; the letters b, c, d and d′ refer to signals of olefin protons. Reprinted with permission from [31]. Copyright (2011) American Chemical Society.

The main types of unsaturated end-groups, related to the desired HRPIB structure and the products of the side processes, can be easily observed using ^1^H NMR spectroscopy [31]. As can be seen in Figure 2, the *exo*-olefinic fragment >C = CH_2_ can be detected through the presence of singlets at δ = 4.68 and 4.89 ppm; the singlet at 5.19 ppm corresponds to the *endo*-olefinic group. The presence of –CMe_2_Cl end-groups can be detected through the signal of Me protons at δ = 1.68 ppm [29].

**Figure 3:**
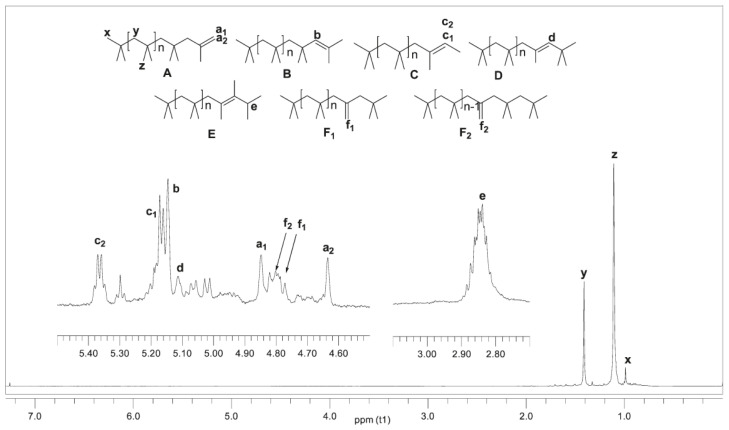
^1^H NMR spectrum of PIB obtained with the H_2_O/FeCl_3_ initiating system. The PIB sample comprises polymers with *exo*- (A) and *endo*-(B) olefinic end-groups, trisubstituted olefinic end-groups (C, D), tetrasubstituted olefinic end-group (E), and internal vinylene isomers (F1, F2). The signals of the end-groups in ^1^H NMR spectra are indicated by corresponding lowercase letters. Reprinted with permission from [32]. Copyright (2011) American Chemical Society.

More detailed analysis of the ^1^H NMR spectra of PIB was carried out by Liu et al. on the product of IB polymerization initiated by H_2_O/FeCl_3_ at 0 °C [32]. The structural types of the olefinic fragments and their assignment are presented in Figure 3. The strong resonance signals at δ = 1.11 ppm (z) and 1.42 ppm (y) were assigned to the –CH_3_ and –CH_2_– protons of the main chain of PIB, respectively; the signal of –C(CH_3_)_3_ head group was observed at δ = 0.99 ppm.

The β-proton abstraction from the growing tertiary carbocation normally leads to *exo*- or *endo*-olefinic end-groups, –CH_2_C(CH_3_)=CH_2_ (A, δ = 4.64 and 4.85 ppm) or –CH_2_CH = C(CH_3_)_2_ (B, δ = 5.15 ppm). The two strong quartet resonances at δ = 5.17 and 5.37 ppm (c1, c2) and the intensive multiple resonances at δ = 2.85 ppm (e) were attributed to –C(CH_3_)=CH(CH_3_) (C for *Z*- and *E*- configuration) and –CH_2_C(CH_3_)=C(CH_3_)CH(CH_3_)_2_ (E) in PIB chains, respectively. The resonances at δ = 4.80 and 4.82 ppm were assigned to internal vinylene isomers F1 and F2, respectively. A weak singlet at δ = 5.12 ppm was attributed to the vinyl proton in –C(CH_3_)=CHC(CH_3_)_2_ (D). In addition, many minor undefined resonances were observed, such as single resonances at δ = 4.77 ppm, doublet resonances at δ = 5.01, 5.03 and δ = 5.06, 5.08 ppm, and triplet resonance at δ = 5.30 ppm.

**Figure 4:**
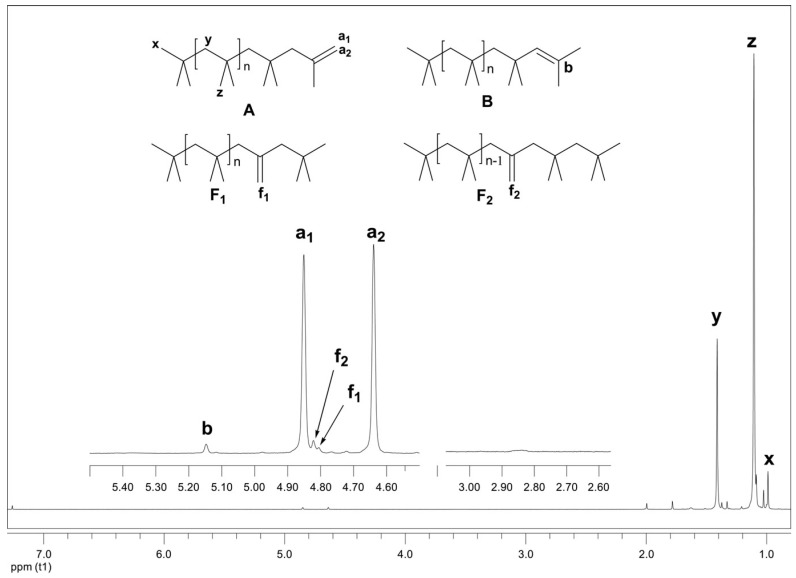
^1^H NMR spectrum of PIB obtained with the AlCl_3_/OBu_2_/H_2_O initiating system. The PIB sample comprises polymers with *exo*- (A) or *endo*-(B) olefinic end-groups, and internal vinylene isomers (F1, F2). The signals of the end-groups in ^1^H NMR spectra are indicated by corresponding lowercase letters. Reprinted with permission from [33]. Copyright (2010) Elsevier B. V.

The formation of F1 and F2 is a substantial side process during the synthesis of HRPIB, and corresponding signals f1 and f2 at δ = 4.82 and 4.80 ppm are more visible in the ^1^H NMR spectra of HRPIB (Figure 4) [33]. These signals can be attributed to the products of 1,3-methide migration (see Section 2.2) or to the protons of =CH_2_ in the coupled PIBs formed by the addition of a growing PIB carbenium ion to the *exo*-olefinic end group in another PIB chain. To determine the real reaction pathway, NMR studies should be accompanied by size exclusion chromatography of PIBs (see Section 2.1.3).

**Figure 5:**
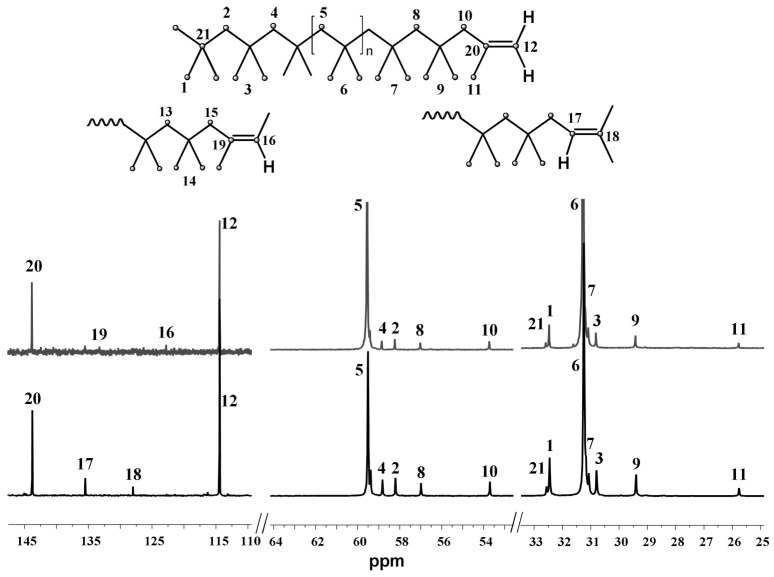
^13^C NMR spectra of HRPIB prepared from the mixed C4 fraction feed (top) and commercial HRPIB (GLISSOPAL 1000, bottom). Reprinted with permission from [34]. Copyright (2013) Wiley-VCH Verlag GmbH & Co.

The ^13^C NMR spectroscopy can also be used for the study of HRPIBs. ^13^C NMR spectra of PIB, prepared using H_2_O/FeCl_3_/^i^PrOH system [34], and commercial GLISSOPAL 1000 are presented in Figure 5.

The ratio of the integral intensities of the end-groups and polymer backbone can be used for the determination of *M*_n_. However, the presence of low-MW products of the chain scission can complicate the analysis of PIB spectra.

#### 2.1.2. Mass Spectrometry

**Figure 6:**
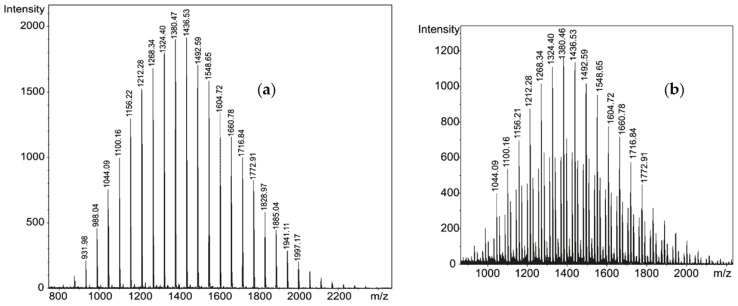
(**a**) Negative-ion APPI-TOF mass spectrum of HRPIB sample from BASF; and (**b**) negative-ion APPI-TOF mass spectrum of PIB sample from Infenium. Reprinted with permission from [31]. Copyright (2011) American Chemical Society.

Mechanistic causes of the formation of tri-substituted olefinic end-groups (see Section 2.2) have been determined using mass spectrometry: appearance of the odd-numbered peaks clearly indicated the possibility of the chain scission during IB polymerization. Dimitrov et al. [31] have analyzed the samples of HRPIB (provided by BASF) and “conventional” PIB with internal double bonds (provided by Infenium) using atmospheric pressure photoionization time-of-flight mass spectrometry (APPI-TOF MS) and have found that only one series of peaks appears in the APPI-TOF mass spectrum of HRPIB (Figure 6a). In APPI-TOF mass spectrum of “conventional” PIB, meanwhile, three additional series of peaks were observed with a mass difference of 14 Da, which corresponds to the –CH_2_– fragment (Figure 6b) [31].

#### 2.1.3. Size Exclusion Chromatography

Size exclusion chromatography (SEC) is a separation technique which is widely used for the determination of molecular weight characteristics of the polymers. Informative and correct studies of the polymers require calibration using polymer standards with known molecular masses and dispersities. However, in everyday practice, such standards are not readily available. Based on universal calibrating principle [35], the relationship between molecular weight *M* and intrinsic viscosity [*η*] is described using the Mark–Houwink Equation (1):

(1) $$[\eta ]=K\cdot M^{\alpha }$$

Reported values of *K* and α value pairs for PIB were quite different. This issue was clarified by the studies of Puskas et al. [36] using PIB samples with *M*_v_ 1–1100 kDa. As a result of the measurements, the values *K* = 2.0∙10^−4^ dL∙g^−1^ and α = 0.67 have been obtained.

In the follow-up work [37], Puskas et al. referred to the problems of the study of branched PIBs, and proposed the use of hyphenated SEC techniques such as SEC coupled with multi-angle laser light scattering (MALLS), viscometry plus universal calibration principle (VIS/UCP), and viscometry plus right-angle light scattering (VIS/RALS). As a result of this study, empirical Equation (2) was established.

(2) $$ML=M_{w}^{0.145}+M_{z}^{-0.02}-20.96,$$

where *ML* is Mooney viscosity, *M*_w_ is number average molar mass, and *M*_z_ is Z-average molar mass of the polymer.

In a recent work [38], fractionations of commercial low-MW HRPIB (*M*_n_ = 902 Da, *Ð*_M_ = 1.8) and “conventional” PIB (*M*_n_ = 880 Da and *Ð*_M_ = 5.1) were performed. “Conventional” PIB contained more low-MW fraction (up to 200 Da), 4.8 vs. 1.3%, and high-MW fraction (>5 kDa), 28.8 vs. 2.4%. Combination of the fractionation with ^1^H NMR analysis of PIB fractions allowed to determine chain-end structures of macromolecules and propose the mechanisms of the formation of different unsaturated fragments.

### 2.2. Mechanism of Isobutylene Polymerization

Isobutylene is capable of being polymerized via cationic [20] and free-radical [39] mechanisms. Free-radical polymerization of IB was carried out previously using *^t^*BuN=N*^t^*Bu initiator in the presence of LiCB_11_Me_12_ with a formation of highly branched PIB [40]. Recently, Hero and Kali [41] reported the results of a study on free-radical polymerization of IB, complexed with cyclodextrin and dissolved in water. Linear PIBs with degree of polymerization (*DP*_n_) > 30 were obtained, and the method was proposed for the synthesis of block copolymers with acrylates. However, PIBs with –CH=CMe_2_ end-groups were formed during free-radical polymerization.

In this way, it can be concluded that the controlled synthesis of linear PIBs with highly reactive end-groups should be based on cationic polymerization, initiated by tertiary carbenium cation [42]. At the subsequent stage, the cationic species react with IB to yield cationic adducts. The chain propagation involves the repetitive addition of isobutylene to the growing carbenium ion until a chain release via chain transfer to IB or other chain transfer agent occurs [23]. It is important to note that the formation of carbenium ions can be accelerated by the addition of Lewis bases; this phenomenon was first observed and explained by Penczek in 1992 [43].

At strong negative temperatures, IB polymerization proceeds as a “living” process, which opens the way for obtaining HRPIBs or functionalized HRPIBs by quenching with different reagents [44]. At subzero and ambient temperatures, cationic polymerization of IB is often complicated by non-selective proton transfer to monomer or chain scission with a formation of >C=CH– fragments, and by rearrangements of carbenium cations with a formation of >C=C< fragments. The mechanism of IB polymerization was studied in detail more than 10 years ago by Dimitrov et al. [31] on the transformations of model PIB-Cl oligomers in the presence of EtAlCl_2_. Matrix-assisted laser desorption/ionization time-of-flight (MALDI-TOF) mass spectrum of the reaction product, obtained at 0 °C, contained two main series of the peaks. The lower intensity series was identical with that of the starting PIB, while the more intense series was 14 Da (mass of one -CH_2_- group) higher and could only form by a loss of C_4*n*−3_ fragments. To prevent reprotonation, ionization of PIB-Cl by EtAlCl_2_ in the presence of 2,6-di-*tert*-butylpyridine (DTBP) was studied at different temperatures using ^1^H NMR spectroscopy (see Section 2.1.1). In the presence of DTBP, *exo*- and *endo*-olefins were formed, and their amount increased with increasing temperature (Table 1). This suggests that the activation energy for proton elimination is higher than that for isomerization/chain scission. In the absence of DTBP, *exo*- and *endo*-olefins were virtually absent at all temperatures, which can be attributed to a preference for reprotonation, subsequent isomerization, and chain scission. Tri-substituted olefins were not observed at 25 °C, which is important for the identification of the mechanism leading to tetrasubstituted olefins.

**Scheme 2:**
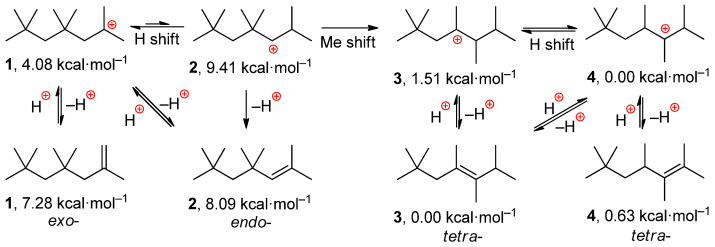
Interconversions and relative stability (*E* scale) of model cations and olefins based on isobutylene trimer [31].

To develop a comprehensive model of the reaction mechanism, the structures of IB trimers and corresponding cations were optimized using the density functional theory (DFT) method (B3LYP/6-31G level of theory, Scheme 2) [31]. Upon ionization, the least stable tertiary cation 1 is formed. A 2-1 hydride shift to form 2 is energetically unfavorable but is not prohibitive. After formation of 2, the system compensates for the loss of energy by further rearrangement via a methyde shift leading to 3, which is the second most stable tertiary cation. As previously reported, the hydride transfer between carbocations 3 and 4 requires very little activation energy [45]. It should be noted that *exo*-olefin is more stable in comparison with *endo*-isomer, i.e., the proton transfer with a formation of *exo*-olefinic group is favorable both sterically and thermodynamically.

**Figure 7:**
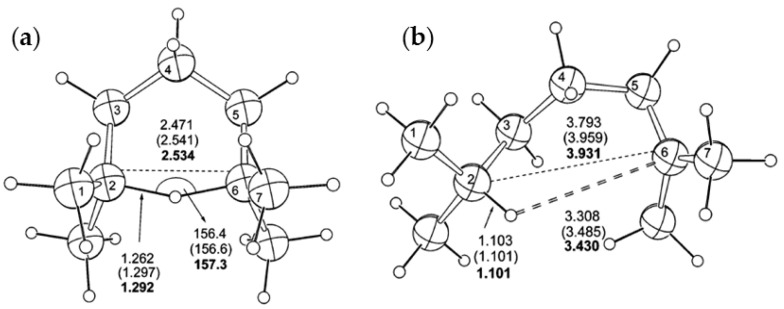
MP2/6-311G(d,p)-optimized geometries of the transition state (**a**) and the key intermediate (**b**) for degenerative 1,5-hydride shift in 2,6-dimethyl-2-heptyl cation (B3LYP/6-311G(d,p) values in parentheses, and the values obtained via B3LYP/6-31G(d) SCI-PCM reaction field model are presented in bold). Reprinted with permission from [46]. Copyright (2006) American Chemical Society.

To determine where the chain scission takes place, partially deuterated model polymers with the composition PhCMe_2_(IB-d_8_)_13_(IB)*_n_*Cl (*n* = 1–6) were synthesized and subjected to ionization with EtAlCl_2_ at −40 °C in the presence of DTBP. ^1^H NMR spectral studies showed that ~3–4 IB units are cleaved during the formation of tri-substituted olefins. Proposed mechanism of the cleavage was based on the assumption that type 4 carbenium ion may have undergone backbiting via a six-membered transition state [31]. Similar reaction pathway was proposed earlier for degenerative 1,5-hydride shift in 2,6-dimethyl-2-heptyl cation (Figure 7); activation barrier for this process was estimated to be ~10 kcal∙mol^−1^ (*E* scale, MP2/6-311G(d,p) level of theory) [46].

**Scheme 3:**
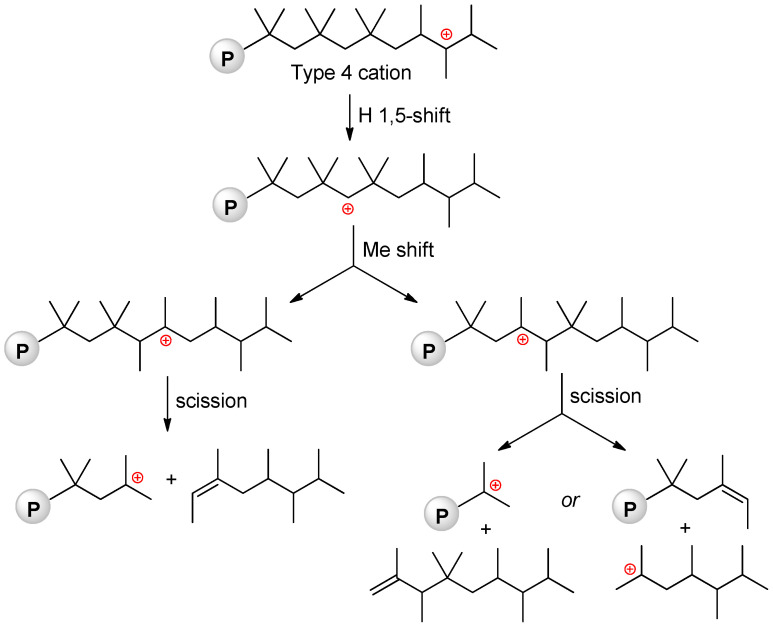
Proposed mechanism of chain scission during isobutylene polymerization [31].

In this way, the reaction mechanism of IB polymerization should be extended to include the chain scission (Scheme 3). In sum, all side products can be correlated with type 3 and 4 cations; at sub-zero temperatures backbiting and chain scission are favored, while at ambient temperatures proton elimination is faster [31].

**Scheme 4:**
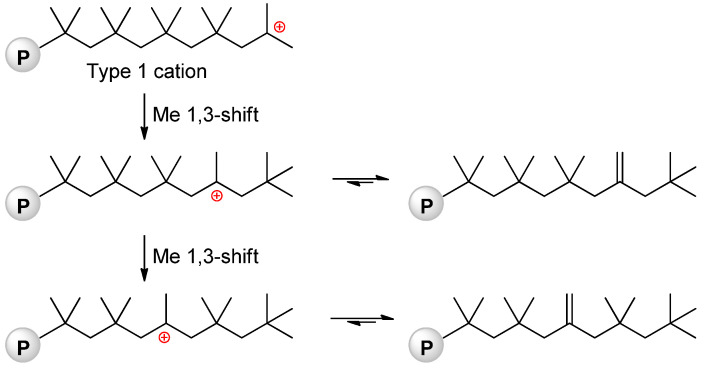
Formation of “internal” *exo*-olefins during cationic polymerization of IB [33].

Cation rearrangements and chain scission processes, discussed above, are more actual for cationic IB polymerization, catalyzed by AlCl_3_/H_2_O and other conventional catalysts unsuitable for the synthesis of HRPIB. During the formation of HRPIB, 1,3-methide shifts become visible (Scheme 4, see also Figure 4).

When using toluene as a solvent, for some catalysts (e.g., ^i^BuAlCl_2_) IB polymerization is complicated by alkylation of the solvent, which leads to the formation of very short PIB chains terminated by a toluene. To clarify the reasons why only very short PIB chains are terminated by alkylate toluene, Kostjuk et al. used the DFT (B3LYP) method to calculate the charges of carbocations formed after protonation of IB (^t^Bu^+^) and also after the addition of 1, 2, 3 and 5 IB molecules; the calculated values of natural bond orbital (NBO) charges were +0.538, +0.526, +0.514, +0.512 and +0.510, respectively [47]. Reducing the charge entails decrease in the reactivity; however, this effect disappears for *DP*_n_ > 5, and cannot be used for the chain control. In 2023, Lee et al. tried to create single-state and multi-state statistical models for cationic polymerization of IB using PIBs with narrow and broad molecular weight distribution (MWD), respectively [48]. Verification of the multi-state model with a chain transfer to proton was performed for AlCl_3_/H_2_O system at 0, 20 and 40 °C with quite good results, despite the debatable assumption about two-stage process of the formation of initiating species (modern ideas about these species are discussed in Section 3.3).

Another industrially important aspect of the chemistry of PIB cations is their reactivity towards other unsaturated hydrocarbons, especially C4 mixed hydrocarbon feeds. De and Faust showed that when using TiCl_4_ catalyst at 0 °C in *n*-hexane media, isobutylene is ~17 times more reactive than buta-1,3-diene, ~543 times more reactive than but-1-ene, and ~294 times more reactive than (*Z*)-but-2-ene. [49]. These conclusions were based on ^1^H NMR identification of the Cl-containing and unsaturated end-fragments formed during capping reactions with C4 hydrocarbons (e. g. (*Z*)-but-2-ene formed –CHMeCHMeCl (~40%), –CMeClEt (~50%) and –CH_2_C(Me)=CH_2_ (~10%) end-fragments). Note that relative reactivity of C4 hydrocarbons in copolymerization with IB correlated with relative reactivities observed in the interaction of C4 olefins with the *p*-methoxy-substituted diphenylmethyl cation in CH_2_Cl_2_ at −70 °C, reported earlier by Mayr [50].

In conclusion of this section, it is worth considering the process opposite to cationic polymerization, viz. enthalpy-driven PIB depolymerization. In the presence of CF_3_SO_3_H, HRPIB depolymerized rapidly at room temperature in aromatic solvents; the process was presumably favored by the formation of stronger C_sp2_−C_sp3_ bonds in *tert*-butylarene in comparison with weaker C_sp3_−C_sp3_ bonds in PIB [51]. This reaction is a particular case of reversible polymerization that has attracted the attention of researchers in recent years [52]; its importance for the synthesis of HRPIB is shaped by the understanding that aromatic solvents can not only terminate cationic polymerization of IB, but also depolymerize the reaction products. That is why IB polymerization should be carried out in bulk or in inert solvents (saturated hydrocarbons, MeCl, etc.).

## 3. Industrial Production of PIBs and Actual Studies of Conventional Catalysts

### 3.1. Industrial Production of PIBs

**Scheme 5:**
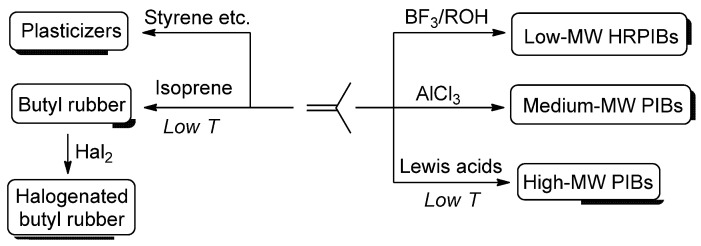
(Co)polymerization of IB and practically important products.

Current technologies of the production of PIBs and their practical applications were considered previously in a number of reviews [1,3,53]. Butyl rubber [42] and halogenated butyl rubbers [1] occupy a significant share of the PIB market; however, the topic of copolymerization of IB with dienes and subsequent chemical modifications are beyond the subject of our review. From the mechanistic point of view and taking into account the catalysts used, the controlled syntheses of low-MW HRPIBs, as well as medium- and high-MW PIBs, are of practical interest (Scheme 5).

Depending on the target MW values and chain-end structure, different catalytic systems and polymerization techniques have been applied in practice. In particular, BF_3_/ROH catalysts are successfully used for the production of low-MW HRPIBs [13,14,15], whereas AlCl_3_/ROH (or H_2_O) and related systems are used for the synthesis of medium-MW polymers [33]. High-MW PIBs dominated the market in 2023 in monetary terms [4], whereas low-MW HRPIBs continue to be the leading volume products, making the study of new efficient catalysts for their synthesis much interesting.

### 3.2. Further Development of BF_3_/ROH Catalysts

Although BF_3_/ROH catalysts have been used in bulk production of HRPIB in recent decades, further studies on the optimization of this system is still underway. Outstanding breakthroughs in this field seem unlikely; however, the results of actual studies in this field have been published. As shown in [54], BF_3_/MeOH can be used as a catalyst for HRPIB production from the technical mixture of C4 hydrocarbons (45 wt% IB; the rest of the components were but-1-ene, but-2-enes and butanes). Polymerization at −20 °C using 0.5 wt% of the catalyst resulted in HRPIB with an *M*_n_ of ~1 kDa. Polymerization of C4 hydrocarbon mixtures containing 36–46 wt% of IB using continuous-flow addition of the BF_3_/MeOH catalyst resulted in HRPIB with *M*_n_ = 0.93–1.23 kDa and an *exo*-olefin content of 71.2–82.3% [55]. Continuous-flow method was also applied for the production of low-MW HRPIB (C8–C28 mixture with 31 wt% of C16) [56].

Polymerization of IB, diluted with an equal amount of butane, with the use of 0.25% BF_3_ and ^i^PrOH (2:3 ratio) at −27 °C yielded HRPIB with *M*_n_ = 2.37 kDa and an *exo*-olefin content of 87.5%; at −17 °C HRPIB, it yielded HRPIB with lower *M*_n_ of 0.96 kDa and an *exo*-olefin content of 93.8% [57]. Further increase in the reaction temperature resulted in a decrease in IB conversion and *M*_n_ content of HRPIB. During polymerization of pure IB, continuous-flow addition of the catalyst resulted in increase in the *exo*-olefin content up to 88% [58].

To produce low-MW HRPIB, the use of IB dimer of the formula *^t^*BuCH_2_C(Me)=CH_2_ as a chain transfer agent and 2,6-di-*tert*-butyl-4-methylphenol as a polymerization-retarding agent were proposed in the patent of the TPG Group [59]. Addition of polymerization-retarding agent resulted in a decrease in the *Đ*_M_ values of PIBs.

For BF_3_-catalyzed processes, IB conversions are typically 75–85%; however, all the efforts to drive the reaction to higher IB conversions reduced the vinylidene content through isomerization. Recycling of unreacted IB requires neutralizing the catalyst and using an impurity adsorption column for removing halogen acid from the distilled material [57]. In this way, monomer recycling implies irreversible consumption of the BF_3_-based catalyst and an accumulation of fluorine- and boron-containing wastewater that later need handling and treatment.

Theoretical study of the reaction of IB with BF_3_–H_2_O complex in *n*-heptane media was conducted recently by Babkin et al. [60] (ab initio, 6-311G** level of theory). They showed that the presence of *n*-heptane molecules leads to a decrease in the activation energy of the initiation stage. However, a visible difference between optimized structures of the reaction products in the absence and in the presence of *n*-heptane (Me_3_C^…^FBF_2_(OH) and Me_2_CCH_2_H^…^O(H)BF_3_, respectively) raises reasonable questions about the reliability of the modeling.

In 2019, mixed catalytic system comprising BF_3_/EtOH and TiCl_4_/H_2_O was studied by Liu et al. [61]. They detected a strong synergistic effect between catalyst components; PIBs with *M*_n_ = 20–60 kDa were obtained.

In conclusion of this section, successful heterogenization of BF_3_-based catalysts should be mentioned. NTP Tec patent [62] describes IB polymerization with the use of calcined (700 °C) mesoporous γ-Al_2_O_3_ or silica-aluminas, treated with BF_3_ or BF_3_/MeOH, respectively. In the first case, polymerization of 63 g of IB using 0.4 g of the catalyst (−5 °C for 40 min) afforded HRPIB with (76% conversion, *M*_n_ = 900 Da, and *exo*-olefin content of 82%). When using silica-alumina/BF_3_/MeOH catalyst (0.2 g) in 45 g of IB polymerized at −5 °C within 20 min with a conversion of 91%, HRPIB with (*M*_n_ = 910 Da and *exo*-olefin content of 80%) was obtained.

### 3.3. AlCl_3_/H_2_O and Related Systems

AlCl_3_-catalyzed polymerization of IB produces conventional PIBs which contain up to 90% of internal double bond end-groups (trisubstituted and tetrasubstituted) [33]. However, since H_2_O can be used as an initiator for the first-generation AlCl_3_-based catalysts, the reaction of H_2_O with AlCl_3_ with the formation of catalytically active species is of particular interest and worthy of separate consideration.

**Scheme 6:**
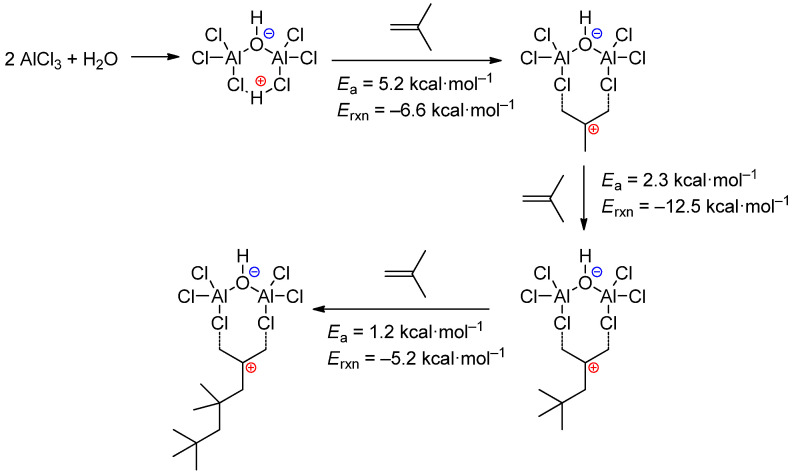
Summary of the proposed initiation and propagation mechanisms with Cl_3_Al(μ-OH)(μ-H)AlCl_3_ [63].

**Figure 8:**
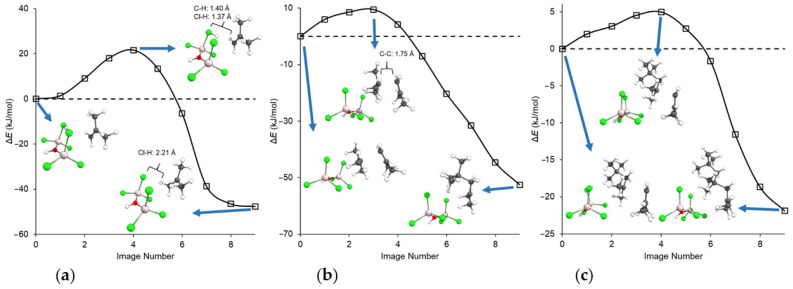
Potential energy surfaces for (**a**) IB initiation with Cl_3_Al(μ-OH)(μ-H)AlCl_3_, (**b**) first propagation stage and (**c**) second propagation stage. Reprinted with permission from [63]. Copyright (2018) American Chemical Society.

The first molecular-level study of the acid-catalyzed IB polymerization was carried out by Johnson’s group in 2018 on the H_2_O/AlCl_3_ system [63]. Generally accepted view on the nature of the catalytic species as AlCl_3_–OH_2_ was not confirmed with DFT calculations (BP86/def2-SVP level of theory; gas phase): the formation of Al_2_ species of the formula Cl_3_Al(μ-OH)(μ-H)AlCl_3_ (Scheme 6) proved to be preferable. In particular, calculated *E*_a_ for the initiation of IB polymerization with AlCl_3_–OH_2_ was 21.6 kcal∙mol^−1^. In contrast, the value of *E*_a_ for Cl_3_Al(μ-OH)(μ-H)AlCl_3_-initiated process was only 5.2 kcal∙mol^−1^, and subsequent stages were found to be exothermic (Figure 8).

Calculations of the solvent effect (CH_2_Cl_2_, conductor-like screening continuum solvation model) resulted in substantial decrease in the activation barriers (*E*_2_ = 3.2 kcal∙mol^−1^ for initiation stage), indicating that Cl_3_Al(μ-OH)(μ-H)AlCl_3_ is likely to be an active catalyst in polar solvents at strongly negative temperatures. The reaction sequence can be attributed to “living” IB polymerization. However, possible reaction pathways of chain release via proton transfer to monomer of elimination with a formation of Cl_3_Al(μ-OH)(μ-H)AlCl_3_ and HRPIB were not analyzed.

An attempt to use the microflow reaction system for AlCl_3_-catalyzed polymerization of IB [64] resulted in obtaining PIBs with *M*_n_ = 20–100 kDa; the content of *exo*-olefinic end-groups in the reaction products was low. In conclusion, it should be noted that AlCl_3_ (or HCl/Et_2_AlCl) was used as a catalyst for low-temperature IB copolymerization with polyfarnesene to obtain highly branched PIBs [65]. The nature of the end-groups was not considered in this patent.

### 3.4. Synthesis of PIB-Cl

In the absence of Brønsted acids, initiation of IB polymerization usually requires the use of *tert*-alkyl chloride (cumyl chloride, *^t^*BuCH_2_CMe_2_Cl, *^t^*BuCl etc) as an initiator [66]. At the same time, in the first study of living IB polymerization, MeC(O)OCMe_2_Ph/BCl_3_ and related systems were used [67]. Commonly, the similar systems invariably consist of two components, namely an initiator (cationogen) and a coinitiator (Lewis acid); their development demonstrates that proper combination of these ingredients is of prime importance in controlled cationic polymerizations [2,23].

**Scheme 7:**
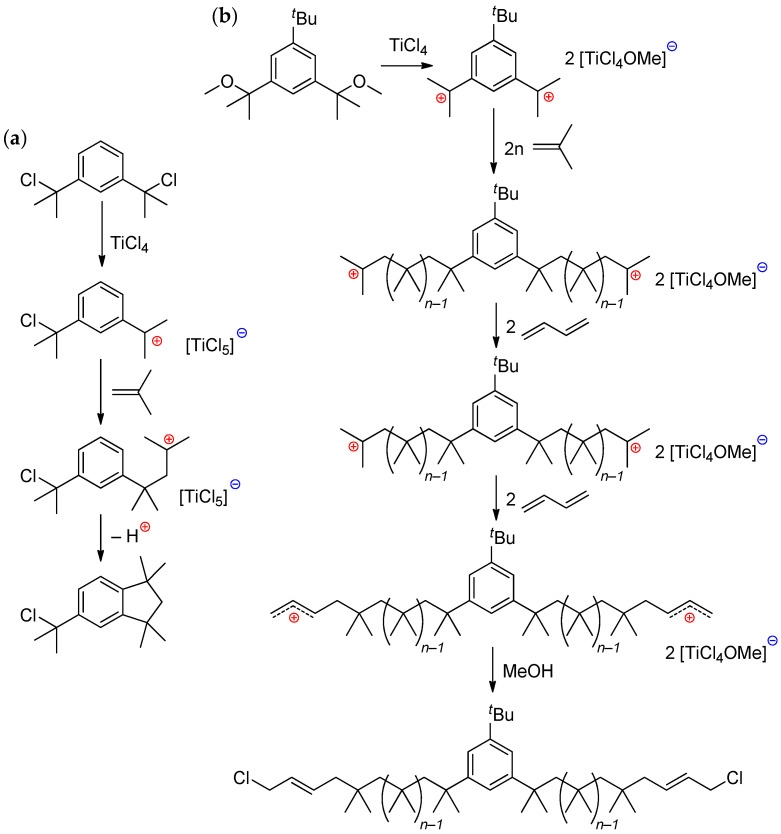
(**a**) Mechanism of the formation of an indanyl ring when using 1,3-bis-(1-chloro-1-methyl-ethyl)-benzene as an initiator; and (**b**) mechanism of the synthesis of Cl–Allyl–PIB–Allyl–Cl in the *^t^*Bu-*m*-DiCuOMe/TiCl_4_ initiating system via carbocationic polymerization in *n*-hexane/MeCl 60/40 (*v*/*v*) at 80 °C [68].

TiCl_4_/*tert*-alkyl chloride have long been successfully used in living low-temperature cationic polymerization of IB. Among recent publications, the study of Wu et al. [68] deserves a separate mention. They have demonstrated that 1,3-bis(1-chloro-1-methyethyl)benzene, a bifunctional initiator commonly used worldwide in carbocationic polymerization, in the case of IB can form indane derivative (Scheme 7a). To avoid this side process, 5-substituted derivative (Scheme 7b), similar to the one previously suggested by Kennedy’s group [69], was proposed. Living IB polymerization was terminated by buta-1,3-diene and MeOH with the formation of PIB-Cl containing highly reactive and easily hydrolyzable CH=CHCH_2_Cl end-fragments.

## 4. Three Generations of Modern Catalysts

### 4.1. First-Generation Catalysts: Metal Chlorides/Ethers

The very idea of developing new polymerization catalysts entails the use of Lewis base as a reaction component. The main potential advantage of these catalytic systems is the ability to conduct IB polymerization at temperatures above zero Celsius with a retention of high *exo*-olefin content in the reaction products. The first group of the catalysts, potentially able to replace BF_3_/ROH systems in the synthesis of HRPIBs, were the complexes of metal halides with ethers.

#### 4.1.1. AlCl_3_/R_2_O and Related Systems

In 2010, Kostjuk et al. proposed a simple and prospective initiating system for the synthesis of HRPIB using cumyl alcohol and AlCl_3_/Bu_2_O [70]; similar type of the initiating system was reported independently by Wu et al. [33]. The most valuable finding of the study by Kostjuk et al. was the gradual decrease in IB conversion and MW of PIB when reaction temperature was increased from −60 to −20 °C [70]. On the other hand, increasing the temperature did not significantly influence the content of *exo*-olefinic end-groups. High activities and regioselectivity were achieved in CH_2_Cl_2_ and toluene solvents, whereas a number of limitations (lower activity and *exo*-olefin content) were observed when *n*-hexane was used as a reaction media [71].

**Figure 9:**
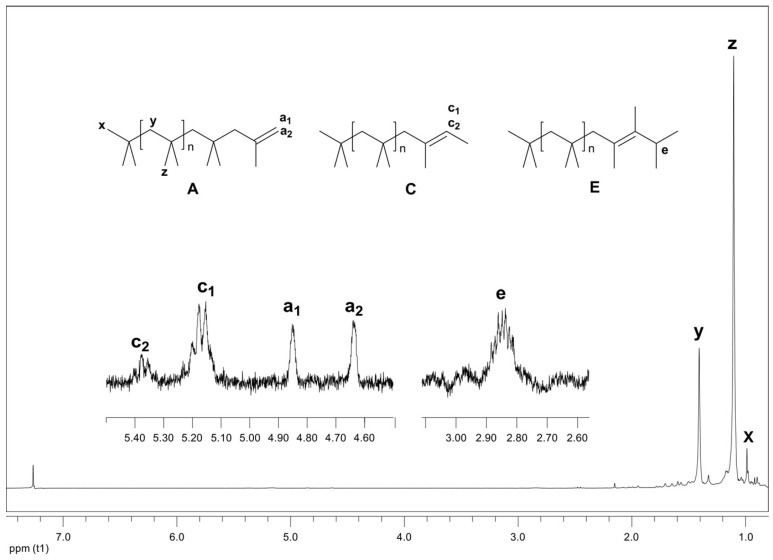
^1^H NMR spectrum (300 MHz) of PIB obtained with the AlCl_3_/H_2_O initiating system. Conditions: [AlCl_3_] = 0.002 M; [IB] = 1.82 M; [H_2_O] = 0.0007 M; CH_2_Cl_2_; T = 0 °C; t = 20 min. The PIB sample comprises polymers with *exo*-olefinic end-groups (A), trisubstituted olefinic end-groups (C), and tetrasubstituted olefinic end-group (E). The signals of the end-groups in ^1^H NMR spectra are indicated by corresponding lowercase letters. Reprinted with permission from [33]. Copyright (2010) Elsevier B. V.

In [33], Wu et al. have clearly demonstrated the marked difference in catalytic behavior of AlCl_3_/H_2_O and AlCl_3_/Bu_2_O catalytic systems. As can be seen in Figure 9, when using AlCl_3_/H_2_O vinylidene route to produce HRPIB is the minor one; tri- and tetra-substituted olefins are the main reaction products.

For AlCl_3_/Bu_2_O catalytic system with H_2_O as an initiator, *exo*-olefinic fragments have proven to be the major end-groups [33]. Apparently, weakly coordinating anion [Bu_2_O^…^AlCl_3_(OH)]^−^ promotes the rapid β-proton abstraction from –CH_3_ of the growing chain-ends with the formation of *exo*-olefinic end-groups and decreases the carbenium ion rearrangements. In addition, the small amount of excess Bu_2_O probably could act as a Lewis base to suppress isomerization. For the AlCl_3_/^i^Pr_2_O system, similar results were reported; the main signals in ^1^H NMR spectrum corresponded to the signals of *exo*-olefinic end-groups (Figure 4). When using the AlCl_3_/Bu_2_O catalytic system, at 0 °C HRPIBs with an *M*_n_ of 1.5–3.3 kDa and an *exo*-olefin content of 76.5–90% were obtained (the latter value depended on IB conversion; at high conversions the vinylidene content decreased). At 20 °C and 45% IB conversion, the *exo*-olefin content was 82%.

Faust et al. [72] have proven that cumyl alcohol is not a true initiator in conjunction with the AlCl_3_/R_2_O catalytic system (this conclusion was drawn based on results of polymerization experiments in the presence of DTP that showed zero activity); the process is initiated with water. Mechanistic studies suggested that the reaction of water with AlCl_3_·R_2_O yields H^+^[AlCl_3_OH]^−^, which initiates the polymerization, and free R_2_O, which abstracts β-proton. In other words, the role of the AlCl_3_·R_2_O complex is to deliver R_2_O molecules to close proximity of the propagating PIB end. In [71], Kostjuk et al. showed that the initiation with water is preferable; when using AlCl_3_∙Bu_2_O/H_2_O, PIBs with 89–94% content of *exo*-olefinic end-groups were obtained in high yield (>85%) within 10 min; *M*_n_ of PIBs obtained was controlled in a range of 1–10 kDa by changing the reaction temperature from −40 to 30 °C.

**Figure 10:**
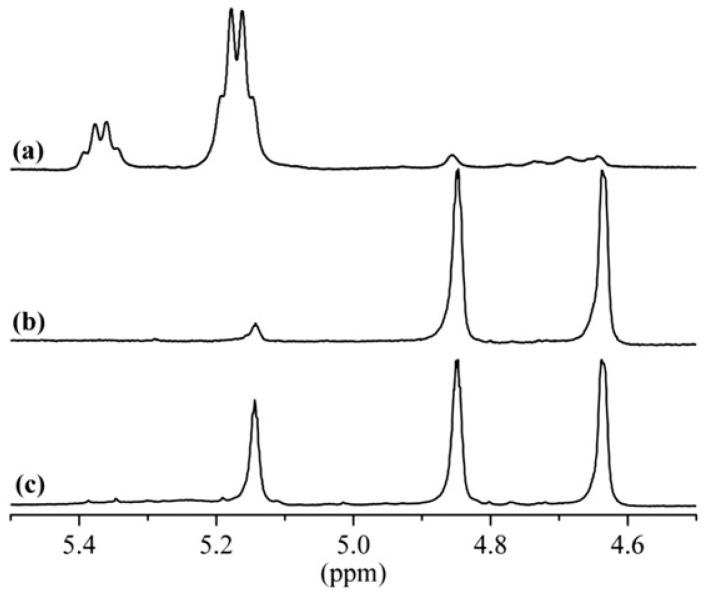
Olefinic region (4.4–5.4 ppm) of ^1^H NMR spectra of PIBs obtained with the AlCl_3_∙Bu_2_O/H_2_O initiating system in toluene at different AlCl_3_/Bu_2_O ratios: [co-initiator] = 22 mM; [IB] = 0.9 M. AlCl_3_/Bu_2_O ratio: (**a**) 1:0.8; (**b**) 1:1; (**c**) 1:1.2. Reprinted with permission from [73]. Copyright (2013) Elsevier B. V.

During the study of IB polymerization, co-initiation by AlCl_3_ complexes with different linear (Et_2_O, Bu_2_O, Am_2_O, Hex_2_O, MeOPh) and branched (^i^Pr_2_O, *^t^*BuOMe) ethers in toluene or CH_2_Cl_2_ at 20 °C was investigated [73]; the complexes with ethers of moderate basicity (AlCl_3_∙Bu_2_O and AlCl_3_∙^i^Pr_2_O) afforded PIBs with the highest *exo*-olefin content (80–95%) and IB conversion, while the use of PhOMe led to the conventional PIBs containing mainly tri- and tetra-substituted olefinic end-groups. The complexes of AlCl_3_ with *^t^*BuOMe and THF were inactive due to the ether cleavage by AlCl_3_. The polymerization of IB in the presence of AlCl_3_∙Bu_2_O or AlCl_3_∙^i^Pr_2_O as co-initiators in *n*-hexane at elevated temperatures (−20 °C to 10 °C) and at high monomer concentrations (up to [IB] ~ 7.8 M) afforded low-MW PIBs (*M*_n_ ~1–8 kDa) with *Ð*_M_ ~ 2, containing 70–85% *exo*-olefinic terminal groups [73]. The 1:1 complexes of AlCl_3_ with linear ethers were found to be stable at ambient temperatures; however, in the case of the AlCl_3_/^i^Pr_2_O system, the formation of less stable and more exchangeable complexes was detected. It is noteworthy that both polymerization rate and chain-end structure of PIB were vulnerable to AlCl_3_/ether ratio: as can be seen in Figure 10, at the ether-starved conditions (AlCl_3_/Bu_2_O = 1:0.8) rapid polymerization of IB took place, and the distribution of end-groups was similar to one obtained with the ether-free initiating system. On the contrary, the use of slight excess of ether (AlCl_3_/Bu_2_O = 1:1.2) resulted in a significant decrease in the reaction rate and *exo*-olefin content, and *endo*-olefin was the main side product. Another significant result reported in [73] was the control of *M*_n_ of PIB within the limits of 3–8 kDa by the length of R in AlCl_3_∙R_2_O.

During further studies of the complexes AlCl_3_∙Bu_2_O and AlCl_3_∙^i^Pr_2_O, IB polymerizations were conducted at −20 to 20 °C in toluene or *n*-hexane media [74]. In both solvents, AlCl_3_∙^i^Pr_2_O showed higher activity and provided higher *exo*-olefin content than AlCl_3_∙Bu_2_O; the presence of small excess of ^i^Pr_2_O over AlCl_3_ (5–10 mol%) resulted in an increase in vinylidene selectivity in some cases. The marked difference between toluene and *n*-hexane was in the effect of temperature on the *exo*-olefin content, which increased in toluene and decreased in *n*-hexane as the temperature increased.

The use of a microflow reactor system for IB polymerization on AlCl_3_∙^i^Pr_2_O within ≤12 s was suggested by Lu et al. [75]. The temperature window was extended from −20 to 50 °C, and the *M*_n_ was adjustable between 0.5 and 15 kDa. However, *exo*-olefin content in the reaction products of the first experiments was 76% or less. Optimization of the reaction conditions in CH_2_Cl_2_ was successful: at 20 °C the selectivity of 89% was achieved with 63% conversion of IB (*M*_n_ = 1.29 kDa, *Ð*_M_ = 1.7); the excess of ^i^Pr_2_O over AlCl_3_ was necessary to avoid the existence of free AlCl_3_ [75]. In further studies of the AlCl_3_∙^i^Pr_2_O system [76], the effect of ethyl benzoate (EB) was evaluated. The reactions at −30 °C were also conducted in a microflow reactor, and PIBS with *M*_n_ = 14.3–47.5 kDa and an *exo*-olefin content of 29–76% were obtained. In more recent works, some aspects of the interactions in AlCl_3_/R_2_O/H_2_O system were detailed [77]. The value of these results for the synthesis of commercially demanded HRPIBs seems questionable.

The use of AlCl_3_∙^i^Pr_2_O as a co-initiator of the polymerization of C4 hydrocarbon feed was studied by Kostjuk et al. [78]. The reaction proceeded at a considerably lower rate in comparison with the polymerization of pure IB yielding PIBs with higher *M*_n_ and *exo*-olefinic end-group content. At 0 °C, *M*_n_ was 4.37 kDa, that is too high value in comparison with *M*_n_ required for practical applications of low-MW HRPIBs (≤2.3 kDa) [12].

Two-component catalytic system, based on AlCl_3_∙^i^Pr_2_O and AlCl_3_∙Et_2_O, was studied recently in *n*-hexane by Lu et al. [79]. In this system, AlCl_3_∙^i^Pr_2_O was confirmed to be the key component that stabilized carbenium ions, and the function of AlCl_3_∙Et_2_O was to promote proton elimination. Polymerizations were conducted in a microflow system with [IB] = 0.33–1.30 M in the presence of H_2_O as an initiator. The yields of IBs were up to 89% after 10 min, and the *exo*-olefin group content was 60–75%.

In recent patents [80,81], the results of the study of AlCl_3_/R_2_O catalyst in IB polymerization (4 M in hexanes) at 0 °C have been presented. As can be seen in Table 2, the use of Bu_2_O instead of ^i^Pr_2_O provided higher *exo*-olefinic content, but lowered the catalytic activity. However, the use of ether mixtures allowed to optimize the process (Table 2, Entry 8); the replacement of the solvent with toluene, as well as Bu_2_O with THF, had no effect.

The further increase in the polymerization temperature to 30 °C for AlCl_3_/R_2_O systems (activated with water in 1:1 [Al]/[H_2_O] ratio) resulted in substantial loss of the selectivity of the formation of *exo*-olefinic groups (12–37%) [82].

The AlCl_3_∙phenetole/TiCl_4_∙H_2_O system was studied by Liu et al. [83]. They found that mixed catalyst exhibited activities 1.2–3 times higher than those for AlCl_3_/phenetole, and more than an order of magnitude higher than those for TiCl_4_/H_2_O, which indicated a notable synergistic effect. However, relatively high-MW PIBs were obtained, and chain-end analysis was not conducted in this work. The results of the study by Deng et al. [84], that describes catalytic experiments with C4 mixed hydrocarbon feed, AlCl_3_/toluene and additional donors (Et_2_O and ethyl acetate), are difficult to analyze because of the absence of data on chain-end structures of relatively low-MW PIBs obtained.

In conclusion of this section, it is appropriate to mention the recent work of the chemists from Beijing University [85] who studied IB polymerization using the AlCl_3_/Bu_2_O catalytic system in a rotating packed bed reactor. The effects of operating parameters including polymerization temperature (T), rotating speed (N) and relative dosage of the monomers and initiating systems ([M]_0_/[I]_0_) on *M*_n_ of HRPIB were studied. Under optimized conditions (10 °C, *N* = 1600 min^−1^ and [M]_0_/[I]_0_ = 49) HRPIB with *M*_n_ = 2.55 kDa and an *exo*-olefin content of 85% was obtained.

#### 4.1.2. Other Metal Chlorides/R_2_O and Related Systems 

The discovery of AlCl_3_/R_2_O catalytic system encouraged the intensive investigations of Lewis acid/ether complexes in the synthesis of HRPIB. In 2011, Wu et al. studied the complexes of FeCl_3_ and a number of ethers (Et_2_O, Bu_2_O and ^i^Pr_2_O) in water-initiated polymerization of IB [32]. In the absence of ethers, conventional non-reactive PIB was formed (see Figure 3 in Section 2.1). Among three ethers, ^i^Pr_2_O demonstrated the highest activity, and the maximum *exo*-olefin content (up to 90% at 0 °C) was observed when using Et_2_O. An increase in [R_2_O]/[FeCl_3_] ratio resulted in a decrease in polymerization rate and an increase in vinylidene selectivity, which generally confirms the possible role of R_2_O in β-proton abstraction proposed by Faust [72].

**Scheme 8:**
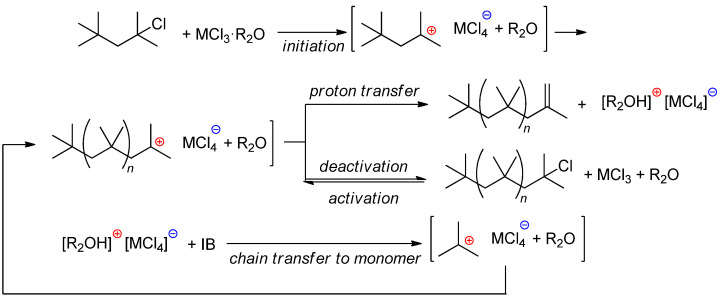
Possible mechanism for the polymerization of IB with the use of MCl_3_∙R_2_O (M = Fe, Ga) and *^t^*BuCH_2_CMe_2_Cl [86].

Up to 85% *exo*-olefin content was achieved in IB polymerization when using MCl_3_∙^i^Pr_2_O (M = Fe, Ga) complexes and *^t^*BuCl or *^t^*BuCH_2_CMe_2_Cl initiators [86]. Di-*n*-alkyl ethers did not form catalytically active complexes. Also of interest was the inertness of AlCl_3_∙^i^Pr_2_O analog in the ionization of *^t^*BuCH_2_CMe_2_Cl. Proposed reaction mechanism is presented in Scheme 8; *^t^*BuCH_2_CMe_2_Cl is ionized by Lewis acid/R_2_O complex to yield *^t^*BuCH_2_CMe_2_^+^, which initiates IB polymerization, and this ultimately results in the release of R_2_O from the complex. The propagating PIB^+^ may undergo proton elimination to yield PIB *exo*-olefin or may be deactivated by ion collapse to yield PIB-Cl, which can be reactivated by Lewis acid. The abstracted proton may then start a new polymer chain after addition to the IB molecule. More detailed kinetic studies of the *^t^*BuCl/FeCl_3_∙^i^Pr_2_O system [87] only confirmed the results of the prior research [86], which is that the use of EtOCH_2_CH_2_Cl and O(CH_2_CH_2_Cl)_2_ instead of ^i^Pr_2_O resulted in a decrease in catalytic activity and *exo*-selectivity.

In 2013, Faust et al. reported the results of a comparative study on the complexation of FeCl_3_ with different ethers and catalytic behavior of the adducts in *^t^*BuCl-initiated IB polymerization [88]. At 0 °C in *n*-hexane ([IB] = 1.0 M, [*^t^*BuCl] = 0.02 M, [FeCl_3_∙R_2_O] = 0.02 M.), 81% conversion and 78% *exo*-selectivity were achieved after 20 min for R = ^i^Pr. However, all experiments with FeCl_3_∙R_2_O in aliphatic media failed to achieve >80% content of *exo*-PIB.

**Figure 11:**
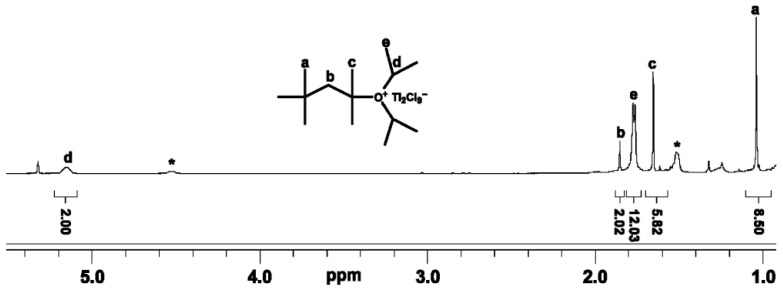
500 MHz ^1^H NMR spectrum of *^t^*BuCH_2_CMe_2_Cl/^i^Pr_2_O oxonium ion adduct in the presence of 10 eq of TiCl_4_ in 50/50 (*v*/*v*) CS_2_/CD_2_Cl_2_ at −70 °C. The lines designated with “a–e” relate to fragments of reactive complex with the relevant designations; lines designated with “*” represent excess ^i^Pr_2_O·TiCl_4_ complex; lines at 1.2 and 1.3 ppm are impurities in CS_2_. Reprinted with permission from [44]. Copyright (2013) American Chemical Society.

The role of the ether molecule in IB polymerization was substantially clarified during the studies of living IB polymerization, initiated by *^t^*BuCH_2_CMe_2_Cl/TiCl_4_ and terminated by ^i^Pr_2_O or *^s^*Bu_2_O with a formation of *exo*-olefinic end-groups after treatment with MeOH [44]. In this study, the formation of PIB–O^i^Pr_2_^+^ ions was proven using ^1^H NMR spectroscopy at −70 °C (Figure 11). When branched ethers were used, 100% *exo*-selectivity was observed; however, experiments with *^t^*BuOMe, Bu_2_O and Et_2_O resulted in the formation of PIBs with 88, 82 and 69% of vinylidene PIB, respectively.

#### 4.1.3. Metal Chlorides/ROH and Related Systems 

**Scheme 9:**
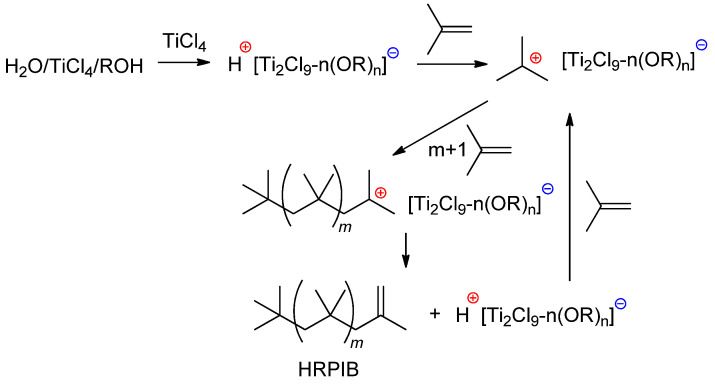
Possible mechanism for synthesis of HRPIB via cationic polymerization of isobutylene with H_2_O/TiCl_4_/ROH (^i^PrOH or ^i^AmOH) initiating system [89].

The possibility of direct synthesis of HRPIB on a H_2_O/TiCl_4_/alcohol initiating system was studied by Wu et al. [89]. IB solution in *n*-hexane and mixed C4 fraction feed ([IB] = 2.9 M) were used in polymerization experiments. H_2_O/TiCl_4_/^i^AmOH initiating system exhibited high selectivity toward the cationic polymerization of IB from the mixed C4 fractions, resulting in the formation of HRPIBs with *M*_n_ = 1.2–1.6 kDa and *Đ*_M_ = 1.5–1.9; vinylidene selectivity was 80%. A possible reaction mechanism (Scheme 9) included the reaction between ROH and TiCl_4_ with the formation of TiCl_3_(OR) and HCl. In the presence of TiCl_4_, protic initiator H[Ti_2_Cl_9-n_(OR)_n_] (n = 0–2) formed and reacted with IB leading to *tert*-butyl cation and PIB. Fast initiation from HCl resulted in higher monomer conversion, lower molecular weight, and narrower MWD of PIBs at higher concentration of ROH. The counter-anion [Ti_2_Cl_9-n_(OR)_n_]^−^ affected the selective β-proton elimination from –CH_3_ groups in the growing PIB chain-ends, and H[Ti_2_Cl_9-n_(OR)_n_] re-initiated IB polymerization.

In addition to etherates, the complex of FeCl_3_ with ^i^PrOH was studied in IB polymerization [34]. When using water as an initiator, this catalyst have demonstrated higher *exo*-selectivity in non-polar solvents, which is distinct from AlCl_3_/R_2_O systems. 

Recently, Kostjuk et al. have studied cationic polymerization of IB with dicumyl chloride/FeCl_3_/^i^PrOH in CH_2_Cl_2_/*n*-hexane 40/60 at −80 °C. They showed that this initiating system afforded bifunctional low-MW PIBs (*M*_n_ 1.25–21 kDa, *Đ*_M_ ≤ 1.28) with –CMe_2_Cl end-groups that can be converted to HRPIBs by end quenching with ^i^Pr_2_O [90].

### 4.2. Second-Generation Catalysts: Alkyl Metal Chlorides/Ethers

#### 4.2.1. Alkylaluminum Chlorides/R_2_O and Related Systems

**Scheme 10:**
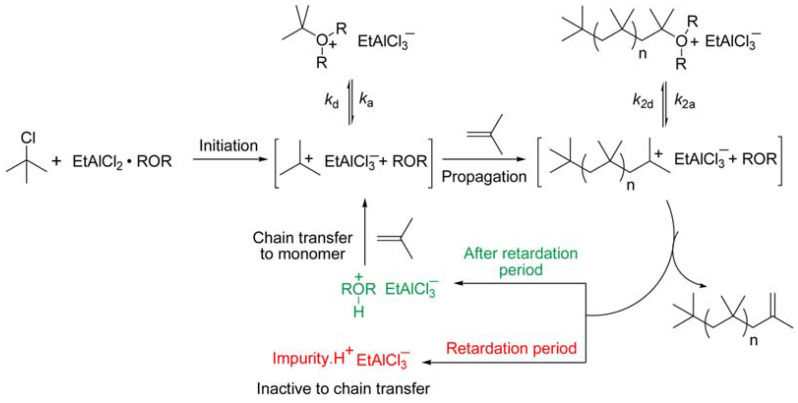
Suggested modified mechanism of IB polymerization in the presence of impurities. Reprinted with permission from [94]. Copyright (2017) Wiley-VCH Verlag GmbH & Co.

When studying the catalytic properties of EtAlCl_2_ in combination with Et_2_O, EtOCH_2_CH_2_Cl and O(CH_2_CH_2_Cl)_2_ in *^t^*BuCl-initiated polymerization of IB, Faust and co-workers have found that only O(CH_2_CH_2_Cl)_2_ is an efficient donor component [91]. At 1:1 [Al]/[ether] ratio and 0 °C, *exo*-olefin content in PIB was 70%, but its value was increased to 90% with the introduction of additional amounts of O(CH_2_CH_2_Cl)_2_ into the reaction. The calculated binding energies of ethers with EtAlCl_2_ (DFT, B3LYP/6-31G* level of theory) were −7.5, −7.1 and −5.1 kcal∙mol^−1^ for Et_2_O, EtOCH_2_CH_2_Cl and O(CH_2_CH_2_Cl)_2_, respectively, which correlates with the ability of EtAlCl_2_ etherate to the displacement with ^t^BuCl with subsequent ionization and polymerization. Depending on the reaction temperature (from −20 to 10 °C), HRPIBs with an *M*_n_ of 1–4.5 kDa were obtained. Another study [92] was focused on the experimental procedure and have only demonstrated the importance of all experimental parameters, including the material of the reaction vessels: when chrome stainless steel reactor was used, *exo*-selectivity of IB polymerization decreased from 90 to ~70%. In the studies of the kinetics and mechanism of IB polymerization, initiated by *^t^*BuCl and catalyzed by EtAlCl_2_·O(CH_2_CH_2_Cl)_2_ in hexanes at 0 °C, Faust et al. explained the first-order kinetic dependence on [IB] concentration and independence of the reaction rate on [EtAlCl_2_]/[O(CH_2_CH_2_Cl)_2_] ratio by postulating an equilibrium between dormant oxonium and active carbenium ions [93]. An increase in the polymerization rate in the presence of water was explained in this work by the influence of water on oxonium/carbenium ion equilibrium. The effect of impurities in the reaction mixture on IB polymerization (Scheme 10) was studied in [94]. In this work, it was shown that low levels of some organic impurities retard the polymerization of IB initiated by *^t^*BuCl and catalyzed by EtAlCl_2_/O(CH_2_CH_2_Cl)_2_ in hexanes at 0 °C. For efficient synthesis of HRPIB, the impurity scavenger Lewis acid needs to be insoluble in hexanes and should not polymerize IB; FeCl_3_ was found to be efficient scavenger.

The EtAlCl_2_/O(CH_2_CH_2_Cl)_2_ initiating system was further optimized by using micromixing module in the polymerization system [95]. HRPIBs with a 100% conversion were obtained within 5 min in the micromixing enhanced system at 20 °C, in comparison to only 30% conversion after 10 min in a conventional reactor. The *M*_n_ and *exo*-olefin content of HRPIBs were adjusted conveniently to the ratio of EtAlCl_2_ to O(CH_2_CH_2_Cl)_2_ and monomer concentration. As a result, HRPIBs with *M*_n_ = 0.8–2.4 kDa, *Đ*_M_ = 2.15–2.85 and *exo*-olefin content of 63–94% were obtained.

**Scheme 11:**
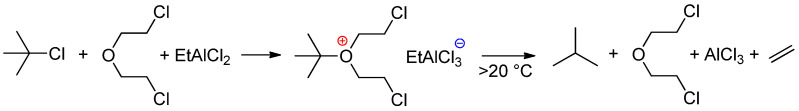
Formation of the oxonium ion from EtAlCl_2_/O(CH_2_CH_2_Cl)_2_ + *^t^*BuCl and its thermal decomposition [96].

Further studies on the EtAlCl_2_/O(CH_2_CH_2_Cl)_2_ initiating system at elevated temperatures showed that the reaction rate could be increased by increasing the polymerization temperature with minimal loss of the *exo*-olefin content [96]. The increase in the polymerization rate was attributed to the increased steady state concentration of carbenium ions; polymerization selectivity depended on the stability of the corresponding *tert*-alkyloxonium cations (resting states of the process, see Scheme 11). The example of *tert*-butyloxonium shows that the oxonium ion is stable up to 15 °C, and slowly decomposes at 20 °C (~15% after 6 min) with the formation of ethylene, isobutane and AlCl_3_ (Scheme 11). This process was clearly confirmed with ^1^H NMR spectroscopy.

**Figure 12:**
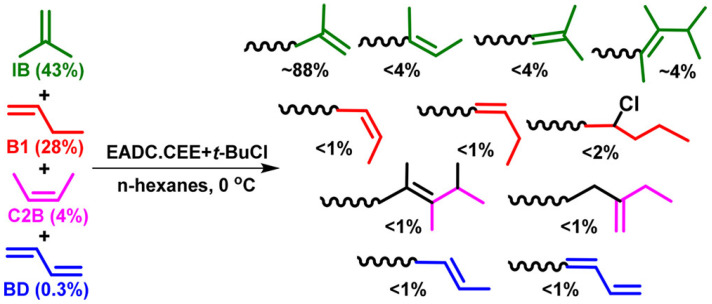
Involvement of C4 unsaturated hydrocarbons in IB polymerization, initiated by ^t^BuCl/EtAlCl_2_/O(CH_2_CH_2_Cl)_2_. Reprinted with permission from [97]. Copyright (2017) American Chemical Society.

From the point of view of industrial applications, the use of C4 hydrocarbon feed instead of pure IB is highly attractive. Faust et al. studied the polymerization of IB in the presence of other C4 olefins, but-1-ene, *cis*-but-2-ene, and buta-1,3-diene using the ^t^BuCl/EtAlCl_2_/O(CH_2_CH_2_Cl)_2_ initiating system in hexanes at 0 °C [97]. As shown in Figure 12, butenes were only marginally involved in copolymerization with IB; buta-1,3-diene was more active, but its involvement was negligible due to its low content in the C4 feed.

IB polymerization using H_2_O and RAlCl_2_∙*n*^i^Pr_2_O (R = Me, Et, ^i^Bu; *n* = 0.6–1) in *n*-hexane at 10 °C was studied by Kostjuk et al. in 2014 [98]. EtAlCl_2_∙*n*^i^Pr_2_O and ^i^BuAlCl_2_∙*n*^i^Pr_2_O (*n* = 0.8–0.9) afforded PIBs with *M*_n_ = 1–1.5 kDa and an *exo*-olefin content of 85–95%. To increase IB conversion and polymerization rate, a “delayed proton abstraction” approach was proposed, i.e., co-initiation by RAlCl_2_ and separately added ether. The most plausible mechanism of the reaction comprised the formation of alumoxanes. The activating effect of water was studied separately [99], and the best results were achieved when ^i^BuAlCl_2_∙0.8^i^Pr_2_O reacted with a suspension of water in *n*-hexane before IB addition. These studies were continued [100], and the conditions of the IB polymerization were optimized by pre-activation of ^i^BuAlCl_2_ using wet argon or MgSO_4_∙7H_2_O in the presence of ^i^Pr_2_O. The highest regioselectivity of polymerization was observed at the ratio [^i^BuAlCl_2_]/[^i^Pr_2_O] of 0.4. The better control of *Ð*_M_ was achieved by using a mixture of ^i^Pr_2_O and Et_2_O as an additive. The optimized conditions were [IB] = 5.8 M, [^i^BuAlCl_2_ preact.] = 38 mM, [Et_2_O] = [^i^Pr_2_O] = 7.6 mM, [H_2_O] = 0.033 M, and 15 min at 10 °C. HRPIBs were obtained with more than 90% yield; they were characterized by an *M*_n_ of ~1 kDa, *Ð*_M_ = 2.4–2.7 and an *exo*-olefin content of >80%. The use of AlCl_3_ or RAlCl_2_ in combination with ethers and mixtures of the ethers was protected by the recent patent [101] (BASF).

In [47], Kostjuk et al. reported on the use of H_2_O/^i^Bu_2_AlCl and H_2_O/^i^BuAlCl_2_·*n*R_2_O (n = 0–1; R_2_O = Bu_2_O, Hex_2_O, ^i^Pr_2_O) initiating systems in the synthesis of medium-MW HRPIBs. The H_2_O/^i^Bu_2_AlCl system initiated slow IB polymerization with a formation of high-MW PIB (up to *M*_n_ = 55 kDa) with a *Đ*_M of_ ~2.5 and an *exo*-olefin content of >85%. Addition of water increased the reaction rate, but almost did not affect the *M*_n_ and vinylidene selectivity. In the absence of ethers, ^i^BuAlCl_2_ catalyzed the formation of ill-defined PIBs and alkylation of toluene (reaction solvent). The addition of the ethers made it possible to control the polymerization; a decrease in R_2_O/^i^BuAlCl_2_ ratio resulted in an increase in IB conversion and *M*_n_ of PIB, and decreased the vinylidene selectivity.

In the recent theoretical work of Zaikov et al. [102], qualitatively different view on the initiation of polymerization of IB with the use of Et_2_AlCl/ROH (R = H, Ph) was proposed. The authors assumed that initiation of the IB molecule proceeds involving EtCl_2_Al^…^O(R)H species, and estimated the activation energies of this process at ab initio HF/3-21G level of theory. Regardless of the modeling results, the very idea of the hydrolytic stability of EtAlCl_2_ is doubtful.

Addition of (Octyl)_3_Al to EtAlCl_2_/O(CH_2_CH_2_Cl)_2_ system resulted in a decrease in the activity with a substantial increase in the *exo*-olefin functionality [103]. Initiation of IB polymerization using this system required *^t^*BuCl or traces the of water; in the latter case, an *exo*-olefin content of 81% was reached [104].

Promising results were obtained when using AlCl_3_/EtAlCl_2_ mixtures [80,81]. As can be seen in Table 3, the low-MW HRPIB with an *exo*-olefin content of more than 80% was formed with high IP conversion in a number of experiments (Table 3, Entries 3, 8 and 13). The synergistic effect, observed in these experiments, still needs further explanation.

#### 4.2.2. Alkoxyaluminum Chloride/R_2_O

**Figure 13:**
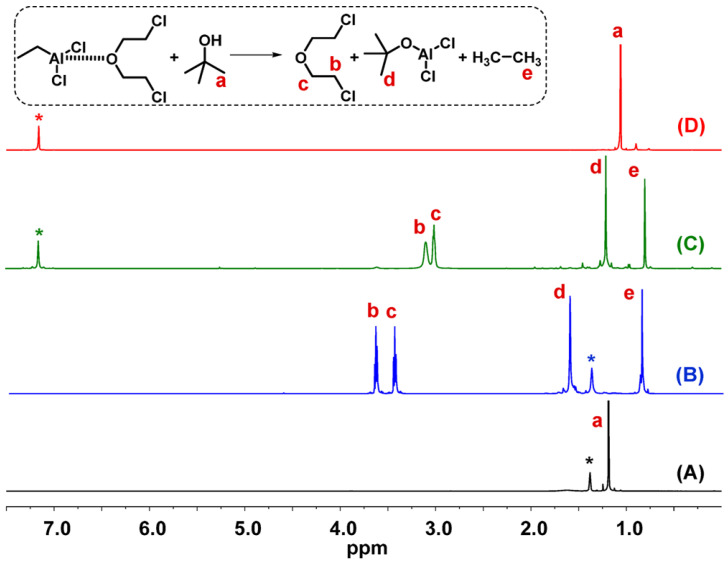
^1^H NMR spectra of (**A**) [*^t^*BuOH] and (**B**) [EtAlCl_2_/O(CH_2_CH_2_Cl)_2_ + *^t^*BuOH] (1:1) in cyclohexane-d_12_ and (**C**) [EtAlCl_2_/O(CH_2_CH_2_Cl)_2_ + *^t^*BuOH] (1:1) and (**D**) [*^t^*BuOH] in benzene-d_6_ at 0 °C. [*^t^*BuOH] = 0.05 M, [EtAlCl_2_/O(CH_2_CH_2_Cl)_2_] = 0.05 M. The lowercase letters denote the structural fragments indicated in the reaction scheme; the asterisk denotes solvent resonance. Reprinted with permission from [105]. Copyright (2018) American Chemical Society.

During the study of the EtAlCl_2_/O(CH_2_CH_2_Cl)_2_ initiating system, Faust et al. have found that an addition of ROH resulted in an increase in vinylidene selectivity [105]. *Tert*-butanol was the most effective “*exo*-enhancer”, that reacted with EtAlCl_2_ to form *^t^*BuOAlCl_2_ species (Figure 13). *^t^*BuOAlCl_2_ is thermally unstable, but can be easily generated in situ. This catalytic system was effective in the polymerization of C4 hydrocarbon feed. Faust et al. suggested that *^t^*BuOAlCl_2_ suppresses the isomerization of PIB^+^ but does not act as a chain transfer agent.

In [106], Kostjuk et al. described the preparation and catalytic study of a number of alkoxy aluminum chlorides. As shown in Table 4, all the alkoxy aluminum dichlorides afforded fast polymerization of IB. However, the *M*_n_ and *exo*-olefin end-group content decreased while polydispersity increased in the series: HexOAlCl_2_ < BuOAlCl_2_ < ^i^PrOAlCl_2_ < PhOAlCl_2_. The decrease in the temperature from 10 to −20 °C (experiments with (BuO)_1.2_AlCl_1.8_) resulted in a significant increase in the *M*_n_ value and vinylidene selectivity. Addition of 0.1 eq. ^i^Pr_2_O to BuOAlCl_2_ resulted in a significant increase in the *exo*-olefin end-group content (up to 90%) with the reduction in *M*_n_ from 5.6 to 2.0 kDa at a high reaction rate (the monomer conversion was completed in 10 min).

**Scheme 12:**
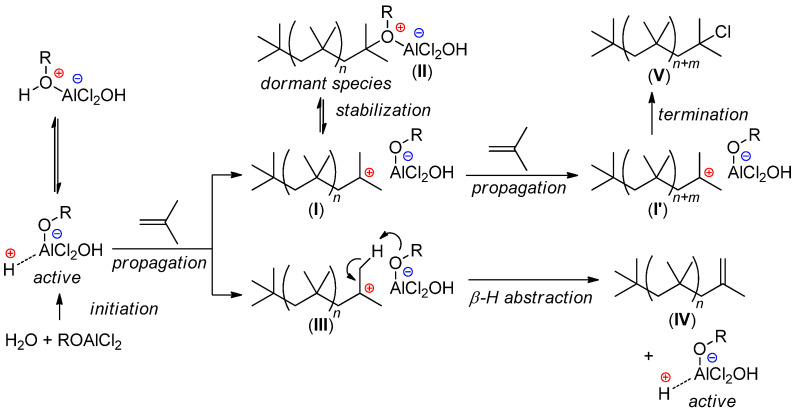
Possible mechanism of the ROAlCl_2_ co-initiated cationic polymerization of IB [106]. The key reaction intermediates and products, discussed in the text, are marked with Roman numerals.

Possible mechanisms of IB polymerization on alkoxy aluminum chlorides is presented in Scheme 12. During the initiation step, ROAlCl_2_ reacts with traces of H_2_O (initiator) to generate a proton, which interacts with a number of IB molecules, highlighting a growing macrocation (**I**). The growing macrocation can be stabilized via the reversible interaction with oxygen of the same or another co-initiator molecule forming a dormant species (**II**). The decrease in reaction temperature results in a shift of equilibrium between the active (**I**) and dormant (**II**) species toward the latter. Similar interaction of the model Ph_2_CH^+^ cation with *^t^*BuOAlCl_2_ was experimentally observed by Faust et al. [105]. This stabilization of the growing species leads to the suppression of the isomerization of macrocation/chain scission that allows for the generation of high molecular weight polymers (I′) in contrast to the bicomponent (Lewis acid/ether) initiating systems. Apart from the stabilization of growing species, ROAlCl_2_ can also regioselectively abstract protons in the β-position to the macrocation (**III**) that results in the formation of *exo*-olefin terminated PIB (**IV**). Termination of polymerization occurs via ion pair collapse with the formation of chlorine-terminated PIBCl.

In a relatively recent BASF patent [107], polymerization of IB was conducted at 10 °C with the use of (BuO)AlCl_2_ as an initiator. When 0.1 eq. ^i^Pr_2_O was added, HRPIB was formed with a high yield (95–99%); *M*_n_ was ~2.0 kDa, and the *exo*-olefin content reached 88–90%. Without ^i^Pr_2_O, the *exo*-olefin content was 46%. Replacement of ^i^Pr_2_O with diethyl ether resulted in a decrease in the catalytic activity.

### 4.3. Third-Generation Catalysts: Heterogeneous Systems

#### 4.3.1. AlCl_3_-Based Ionic Liquids

In 2016, Kostjuk et al. [108] suggested the use of ionic liquids (ILs), prepared from AlCl_3_ and 1-ethyl-3-methylimidazolium chloride (emim-Cl) in combination with ^i^Pr_2_O, for the synthesis of HRPIBs with more than 90% *exo*-olefin content. In the absence of donor, ILs with χ(AlCl_3_) = 0.6 catalyzed the formation of low-MW PIBs (*M*_n_ = 1.36 and *Ð*_M_ = 6.8, Entry 1 in Table 5) containing tri- and tetra-substituted olefinic end-groups. When 0.5 mol equivalents of ^i^Pr_2_O were introduced, well-defined PIBs with a high *exo*-olefin content and a narrow MWD was formed (Entry 2 in Table 5). A number of additional experiments were also conducted. Their results are also presented in Table 5. Since IL-based catalyst is heterogeneous, ultrasonification was used to increase the reaction rate and IB conversion. There was no significant influence of the 3 min ultrasonification before polymerization on the content of the *exo*-olefinic groups; when the conversion reached 95%, *M*_n_ increased.

**Figure 14:**
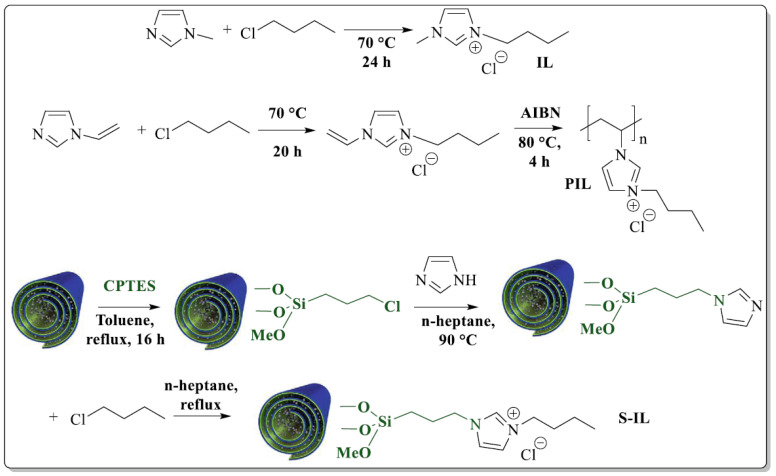
Employed procedure for the synthesis of IL, PIL and S-IL. Reprinted with permission from [109]. Copyright (2022) Elsevier B. V.

The scientific group of Bahri-Laleh has also conducted studies on ILs in the polymerization of IB [109]. They selected 1-butyl-3-methylimidazolium chloride (bmim-Cl) as a reference system, and prepared a polyionic liquid (PIL) via radical polymerization of 1-butyl-3-vinylimidazolium chloride, and a halloysite clay (Hal) supported ionic liquid (S-IL) (Figure 14). With the use of EtOH as an initiator, a number of polymerization experiments were conducted at −20 and 0 °C. PIBs with *M*_n_ = 1.5–11.6 kDa were obtained; however, the *exo*-olefin content in all the samples was less than 19%. Nevertheless, such products are also in demand, and the practical use of ILs without additional donors cannot be ruled out. These studies were continued with the use of boehmite as a support for ILs and the use of C4 hydrocarbon feed as a raw material [110]. The content of isobutylene units in the polymer was 75–85%, with low *exo*-olefinic content. It should be noted that the use of boehmite-based S-ILs allowed to halve AlCl_3_ loading in comparison with the use of neat AlCl_3_, while maintaining the same PIB characteristics, even more so; the *exo*-olefinic content in the first case was lower. Given that the resulting PIBs were intended for use as oil viscosity improvers, lowering the *exo*-olefin group content should result in an increase in the thermooxidative stability of these additives (see also Section 5.5).

AlCl_3_/urea/arene ionic liquids were proposed to be used in IB polymerization; the ratio AlCl_3_/urea was 2:1 [111]. Polymerization experiments were conducted at 10 °C using 0.3 wt% of the ionic liquid suspensions; when using C_6_H_6_ as an aromatic solvent, the *exo*-olefinic selectivity amounted to 76%. This patent application contains no data on the molecular weight characteristics of the polymers obtained.

#### 4.3.2. Other Ionic Liquids

To solve the problem of slow polymerization with the emimCl–AlCl_3_ initiating system, Kostjuk et al. have studied the influence of ionic liquid acidity, and the nature of Lewis acid and imidazolium salt on the reaction rate [112]. When comparing ILs prepared from AlCl_3_, AlBr_3_, ^i^BuAlCl_2_, FeCl_3_, GaCl_3_ or BBr_3_ and emim-Cl, they showed the highest activity and vinylidene selectivity for the AlCl_3_-based ILs in the presence of 0.5 equivalents of ^i^Pr_2_O. Polymerization was slower with emim-Cl–FeCl_3_ and emim-Cl–GaCl_3_, while the content of *exo*-olefin groups almost does not change with the monomer conversion. The use of emim-Cl–FeCl_3_ and emim-Cl–GaCl_3_ allowed to synthesize HRPIBs with *M*_n_ = 1.7–2.0 kDa and *Đ*_M_ = 1.9–2.2, whereas *M*_n_s of the PIBs obtained with emim-Cl–AlCl_3_ were slightly higher than required (>3 kDa). Ultrasonification was highly effective for emim-Cl–FeCl_3_, after 3 min of pre-treatment; for [IB] = 5.2 M in hexane, almost full monomer conversion was achieved after 20 min at 0 °C with the formation of an HRPIB with (*M*_n_ = 1.9 kDa, *Đ*_M_ = 2.3, and *exo*-olefin content of 82%). Taking into account all the parameters, emim-Cl–FeCl_3_ was considered as the most promising catalyst among the others.

The effect of *^t^*BuCl on the activity of the emim-Cl–FeCl_3_/ ^i^Pr_2_O (2:1) system in IB polymerization manifested as a decrease in the *M*_n_ and *Đ*_M_ values and an increase in vinylidene selectivity [113]. Addition of the minimal amounts of aromatic hydrocarbons (1 vol%) resulted in an increase in the polymerization rate in the following order: benzene < toluene ≈ mesitylene.

The study of the emim-Cl–FeCl_3_/ ^i^Pr_2_O (2:1) system in the polymerization of C4 mixed hydrocarbon feed [78] showed a marked difference from pure IB polymerization. Firstly, a high monomer conversion was obtained for IB polymerization at [IL] = 33 mmol·L^−1^, while in the case of C4 mixed feed polymerization, a higher concentration of ionic liquid was required. Secondly, relatively high molecular weight polymers were formed during the emim-Cl–FeCl_3_-co-initiated polymerization of C4 mixed feed at high co-initiator concentrations as compared to the polymerization of IB. Also, independent of the nature of the monomer used (IB or C4 mixed feed), PIBs had low polydispersities (*Ð*_M_ < 2.8). The use of ultrasonification resulted in an increase in the conversion, and HRPIBs with *M*_n_ = 2.5 kDa, *Ð*_M_ = 2.5 and an *exo*-olefin content of 81% was obtained from the C4 feed.

1-Butyl-3-methylimidazolium (bmim)-based ILs with different counterions in combination with TiCl_4_ have been studied by Wu et al. [114]. [bmim][PF_6_] was found to be the best when using the TiCl_4_/H_2_O initiating system, and HRPIBs with *M*_n_ = 1.0–3.7 kDa, *Ð*_M_ = 1.9–4.4 and an *exo*-olefin content of 49.6–87.4% were obtained in CH_2_Cl_2_ at −10 °C. When using BCl_3_ instead of TiCl_4_, vinylidene selectivity decreased.

In the recent work [115], Kostjuk et al. reported the results of a study on silica-supported emim-Cl/FeCl_3_ ILs of different compositions. They showed that supported ILs with a 30 wt% emimCl/1.5FeCl_3_ content possessed a higher specific surface area and demonstrated high activity and regioselectivity in the cationic polymerization of IB in *n*-hexane, affording HRPIBs with *M*_n_ < 1.5 kDa (*Đ*_M_ < 2) and 70–90% content of *exo*-olefinic end-groups in a range of [IB] = 1–5 M.

#### 4.3.3. Liquid Coordination Complexes

Liquid coordination complexes (LCCs) along with acidic ILs represent a new class of liquid Lewis acids. They usually consist of metal halides (AlCl_3_, GaCl_3_, TiCl_4_, etc.) and donor molecules (e.g., phosphine oxides). In 2021, Kostjuk et al. reported the results of the studies on the preparation of phosphinoxide-based LCCs and their catalytic behavior in IB polymerization [116]. For the Oct_3_PO/AlCl_3_ (χ(AlCl_3_) = 0.60) system in the presence of ^i^Pr_2_O, PIBs with moderate to low *exo*-olefin content (48, 23 and 2%) were obtained at different temperatures (0, 10 and 20 °C, respectively); *M*_n_ values were 3.6–6.5 kDa. When using EtOCH_2_CH_2_Cl, *M*_n_ values were 2.0–5.0 kDa with a vinylidene selectivity of ~65% for all three temperatures. In the presence of O(CH_2_CH_2_Cl)_2_ at 0 °C, HRPIBs with *M*_n_ = 6.9 kDa was obtained and the *exo*-olefin content was 81%; the polymer contained 19% of –CMe_2_Cl end-groups. Further experiments with the latter system allowed to optimize the process conditions, and at 20 °C, an HRPIB with *M*_n_ = 2.2 kDa, *Đ*_M_ = 1.9 and an *exo*-olefin content of 90% was synthesized.

**Figure 15:**
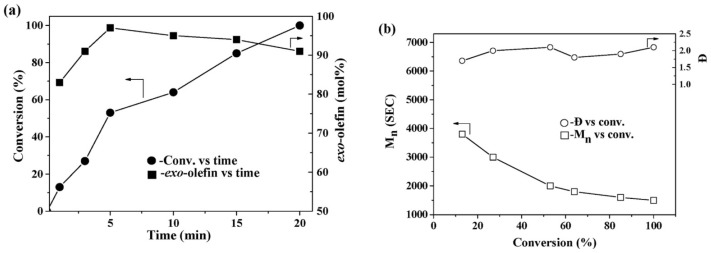
(**a**) Conversion, *exo*-olefin end-group content versus time and (**b**) *M*_n_, *Đ*_M_ versus conversion dependences for the cationic polymerization of IB catalyzed by Oct_3_PO–FeCl_3_/^i^Pr_2_O initiating system: χ(FeCl_3_) = 0.60; [Oct_3_PO–FeCl_3_] = 22 mM, [^i^Pr_2_O] = 11 mM; [IB] = 5.2 M; T = 0 °C. Reprinted with permission from [116]. Copyright (2021) Elsevier B. V.

For FeCl_3_-based LCCs, the effect of additional ether was markedly different; ^i^Pr_2_O has been proven to be the best donor component. At 0 °C, HRPIBs with *M*_n_ = 1.5–2.1 kDa and an *exo*-olefin content of 91–97% were obtained. The polymerization proceeded smoothly and relatively fast (Figure 15): about 50% of monomer conversion was reached in 5 min and 100% in the following 15 min. It is interesting to note that at low conversion (17%), a relatively low *exo*-olefin end-groups content was observed due to the presence of a significant fraction of PIBCl. The PIBCl chains completely disappeared in the course of polymerization (no PIBCl chains at 53% conversion) due to their ionization by chlorophilic Oct_3_PO–FeCl_3_ followed by regioselective β-H elimination with the formation of *exo*-olefinic end-group.

**Figure 16:**
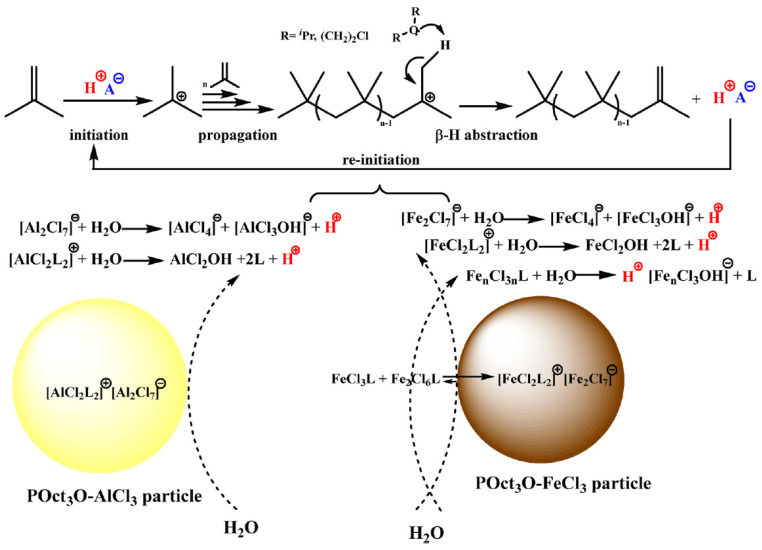
Proposed mechanism for cationic polymerization of IB in *n*-hexane catalyzed by liquid coordination complexes (Oct_3_PO–AlCl_3_ and Oct_3_PO–FeCl_3_) in the presence of ethers (L = Oct_3_PO; A^−^ = M_n_Cl_3n+1_^−^ or M_n_Cl_3n_OH^−^, where M = Al or Fe). Reprinted with permission from [116]. Copyright (2021) Elsevier B. V.

Based on the results of catalytic experiments and UV-Vis spectral data, the mechanism of cationic polymerization of IB catalyzed by liquid coordination complexes (Oct_3_PO–AlCl_3_ and Oct_3_PO–FeCl_3_) in *n*-hexane was proposed (Figure 16). As in the case of ILs, the reactions occurred heterogeneously at the interface of the catalyst particles, with adventitious water acting as an initiator. In contrast to imidazole-based ILs bearing non-reactive imidazolium cation, LLCs contain electron-deficient metal centers stabilized by two ligands ([MCl_2_L_2_]^+^, M = Al or Fe). Therefore, the formation of superacidic protons more likely proceeds via partial hydrolysis of both cationic and anionic parts of LCCs. This assumption correlates well with the much higher activity of LLCs observed in comparison with the corresponding ILs. In addition, partial dissolution of Oct_3_PO–FeCl_3_ could not be excluded, and is supported by bimodal MWD of the PIB. In contrast to Oct_3_PO–FeCl_3_, Oct_3_PO–AlCl_3_ only heterogeneously catalyzed the polymerization of IB at the catalyst particle interface.

#### 4.3.4. Silica-Supported Catalysts

As disclosed in the Braskem S.A. patent [82], the EtAlCl_2_/^i^Pr_2_O catalyst can be supported on silica, 3-aminopropyl-functionalized silica, or ZrO_2_; the obtained catalysts retained high selectivity in the formation of *exo*-olefinic group up to 82–85% even at 30 °C. The catalytic activities were 28–55 μmol_IB_∙mol_Al_^−1^∙s^−1^. Evaluation of the prospects of similar catalytic systems is hampered by the lack of data on molecular weight characteristics of the PIBs obtained.

## 5. Other Catalysts and Methods

### 5.1. Bis(Arene) Metal Cationic Complexes

The complexes of Ga(I) of the formula [Ga(arene)_2_]^+^[Al(OR^F^)_4_]^−^ (arene = C_6_H_5_F, 1,3,5-Me_3_C_6_H_3_, OR^F^=OC(CF_3_)_3_) catalyzed the polymerization of IB in CH_2_Cl_2_ or toluene media with the formation of low-MW HRPIB [117]. At 10 °C in toluene, a polymer with an *M*_n_ of ~2 kDa and an *exo*-olefin content of 93% was obtained using 0.007 mol% of the complex [Ga(C_6_H_5_F)_2_]^+^[Al(OR^F^)_4_]^−^. Two different mechanisms, a cationic-initiated chain growth polymerization in CH_2_Cl_2_ and a coordinative polymerization in toluene, were proposed. In the latter case (toluene media), the Ga(I) salt can be seen as a relatively stable precursor for cyclogalla(III) cations which, being much more electrophilic than Ga(I), initiate and catalyze the polymerization of IB with high reactivity. In further studies [118], bis(arene) *ansa*-ligand PhCH_2_CH_2_Ph and 1,3-Ph_2_C_6_H_4_ were proposed for Ga(I) complexation. IB polymerization experiments (toluene) using [Ga(PhCH_2_CH_2_Ph)_2_]^+^[Al(OR^F^)_4_]^−^ and [((C_6_H_5_F)Ga(1,3-Ph_2_C_6_H_4_))_2_]^2+^[Al(OR^F^)_4_]_2_^−^ afforded PIBs with *M*_n_ = 0.94–2.92 kDa and an *exo*-olefin content of 87–93%. The latter complex was more active at 5 °C in comparison with the former; however, the catalytic activity of [Ga(C_6_H_5_F)_2_]^+^[Al(OR^F^)_4_]^−^ was higher in comparative experiments.

**Figure 17:**
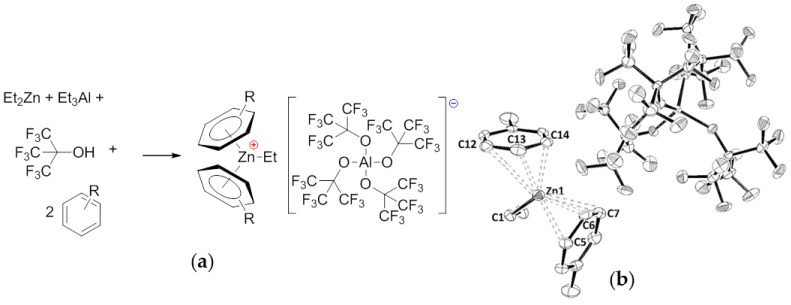
(**a**) Synthesis of [EtZn(arene)_2_]^+^[Al(OR^F^)_4_]^−^ complexes; and (**b**) molecular structure of the [EtZn(PhMe)_2_]^+^[Al(OR^F^)_4_]^−^ complex. Reprinted with permission from [119]. Copyright (2016) Wiley-VCH Verlag GmbH & Co.

In 2016, Krossing et al. tried to obtain the ionic compound [RZn]^+^[Al(OR^F^)_4_]^−^, but failed [119]. The reactions of EtZn(Al(ORF)_4_) with toluene, mesitylene, or o-difluorobenzene yielded [EtZn(arene)_2_]^+^[Al(OR^F^)_4_]^−^ salts (Figure 17a) that were found to be active in IB polymerization. However, low-MW HRPIB was obtained using the [EtZn(PhMe)_2_]^+^[Al(OR^F^)_4_]^−^ complex (Figure 17b), and only moderate conversions (up to 33%) were achieved. At 15 °C, the *exo*-olefin content was 84%.

**Figure 18:**
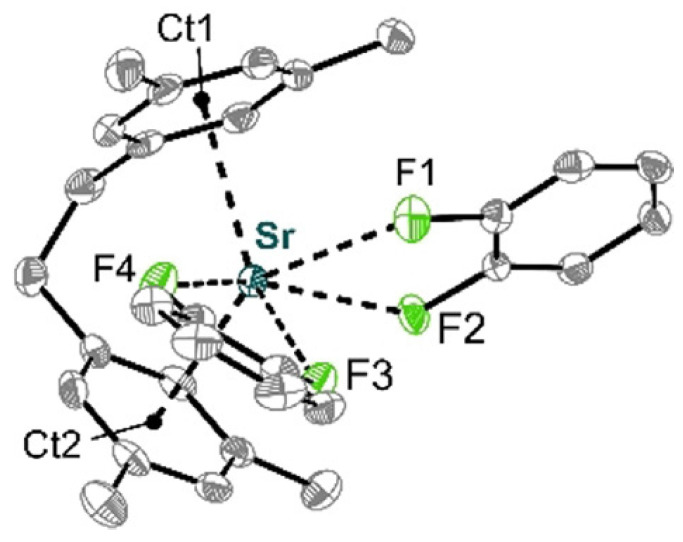
Molecular structure of the dicationic moiety in [Sr(DXE)-(o-DFB)_2_]^2+^ ([Al(OR^F^)_4_]^−^)_2_. Thermal displacement of ellipsoids set at 50% probability. Protons and counterions were omitted for clarity. Reprinted with permission from [120]. Copyright (2020) Wiley-VCH Verlag GmbH & Co.

Subsequent studies of the same scientific group were focused on highly acidic dicationic strontium complex, based on the bis(arene) *ansa*-ligand [(3,5-Me_2_C_6_H_3_)CH_2_]_2_ with additional 1,2-difluorobenzene coordination and [Al(OR^F^)_4_]^−^ counterions [120]. This complex (Figure 18) readily polymerized the IB at 0 or −12 °C, forming PIBs with *M*_n_ = 70 and 130 kDa, respectively. The data on chain-end structure of the PIBs are not mentioned in this paper.

### 5.2. Perfluorophenyl Metal Derivatives

The initiation of IB polymerization with Zn(C_6_F_5_)_2_ and *^t^*BuCl resulted in high-MW PIBs; the catalytic activity of this system was low [121]. However, Zn(C_6_F_5_)_2_ is a convenient starting compound for the synthesis of perfluorophenyl derivatives of other metals, e.g., Al(C_6_F_5_)_3_ and Ga(C_6_F_5_)_3_ [122]. These complexes were studied as catalysts of IB polymerization. In the toluene/saline reaction media, high-MW PIBs with ~1:1 ratio of the *exo*- and *endo*-olefinic end-groups were obtained [122]. Experiments in toluene and hexane, as well as bulk polymerization, also resulted in high-MW PIBS containing <50% of vinylidene end-groups [123].

In a recent study [124], Cai et al. showed that IB polymerization in the temperature interval from −60 to 15 °C in a non-halogenated solvent using Al(C_6_F_5_)_3_ as a Lewis acid and PhCMe_2_Cl as an initiator resulted in PIBs with medium-molecular weights (9–148 kDa). Addition of the donor (Ph_2_O) resulted in the formation of highly active catalysts; however, the *M*_n_ value of the PIB, synthesized at −40 °C, amounted to 355 kDa. Evidently, further studies of similar complexes with regard to HRPIB synthesis have limited chances of success.

**Scheme 13:**
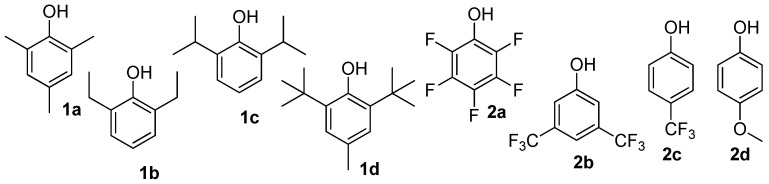
Structures of phenols studied in cationic polymerization of IB initiated by phenols/Al(C_6_F_5_)_3_ systems [125]. Molecular structures of the phenols are indicated by the numbers used in Table 6 for comparison of the catalysts.

When phenols (Scheme 13) were used in combination with Al(C_6_F_5_)_3_ in IB polymerization in toluene, medium-MW PIBs were obtained [125]. The best results were sterically achieved with hindered 2,6-di-*tert*-butyl-4-methylphenol (BHT) and 3,5-bis(trifluoromethyl)phenol (Table 6).

### 5.3. Metal Trifluoromethylsulfonates

Commercial trifluoromethylsulfonates of Sc(III) and Yb(III) were studied by Lewis and Damodaran in IB polymerization using different initiators (H_2_O, MeOH, AcOH, and CF_3_COOH) [126]. Yb(OTf)_3_ with MeOH catalyzed the formation of low- and medium-MW PIB with broad MWD (*M*_w_ = 7.0–25.7 kDa and *Đ*_M_ = 1.97–11.3) and 85–97% vinylidene selectivity; however, polymerization results were poorly reproducible. The use of Sc(OTf)_3_ allowed to obtain low-MW HRPIBs (*M*_w_ = 0.97–2.19 kDa and *Đ*_M_ = 2.0–3.5) with a high *exo*-olefin content (71–88%). Given the high load (~1 wt%) and rather high price of the metal triflates, the prospects of using similar catalysts in practical applications seem questionable.

### 5.4. Visible Light-Induced Polymerization

**Scheme 14:**
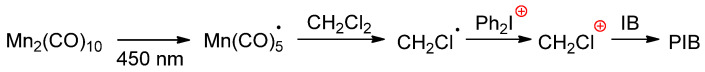
Tentative mechanism of photo-initiated cationic polymerization of IB [129].

Photo-induced controlled/living polymerization has been intensively investigated in recent years [127,128]. Interesting results of the first study on photo-induced cationic IB polymerization were presented in 2023 by Kostjuk at the 14th APME conference [129]. Using the PhCH_2_Br/Mn_2_(CO)_10_/[Ph_2_I]^+^[PF_6_]^−^ initiating system, they have demonstrated that during the photoinitiated cationic polymerization of IB, PhCH_2_Br alone is inactive, while initiation occurred via a chlorine radical abstraction from the solvent (CH_2_Cl_2_), followed by the oxidation of generated radical to the corresponding cation by [Ph_2_I]^+^ (Scheme 14). According to this scheme, the role of PhCH_2_Br is unclear. The reaction proceeded with an induction period at −30 °C in CH_2_Cl_2_/*n*-hexane, affording an *exo*-olefin terminated PIB (*exo*- content >85%) with an *M*_n_ up to 3 kDa and a *Ð*_M_ of < 1.7. The *M*_n_ could be efficiently controlled from 2 to 30 kDa with the concentration of [Ph_2_I]^+^[PF_6_]^−^.

### 5.5. Synthesis of PIBs with Higher Content of Trisubstituted Olefinic End-Groups

**Scheme 15:**
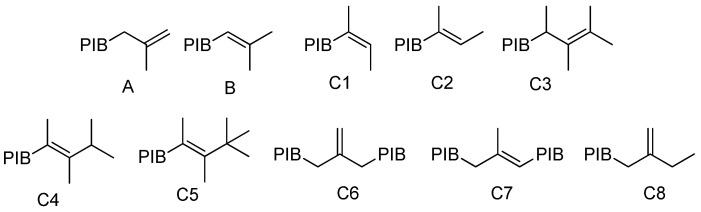
Structures of PIBs in starting HRPIB (A is the main component) and in isomerization products [130]. Molecular structures are indicated by the symbols used in Table 7.

High levels of control molecular weight and vinylidene content in modern HRPIB technologies have the opposite effect. Due to lower photooxidative stability of vinylidene olefins, it became necessary to develop approaches to PIBS with a high content of trisubstituted olefinic end-groups. In the recent BASF patent [130], the results of isomerization of commercial HRPIB Glissopal 1000 in the presence of Al_2_O_3_ and molecular sieves were presented. The course of the reaction is illustrated through the example presented in Scheme 15 and Table 7. This study is of particular interest in terms of isomerization of HRPIBs in the presence of weak acids. It is obvious that such side reactions can proceed during any cationic polymerization of IB.

## 6. Further Transformations of HRPIB (PIB-Cl) and Applications of the Products

### 6.1. Recent Studies on the Synthesis of PIB-SA and PIB-SI

#### 6.1.1. Synthesis and Structure of PIB-SA

**Scheme 16:**
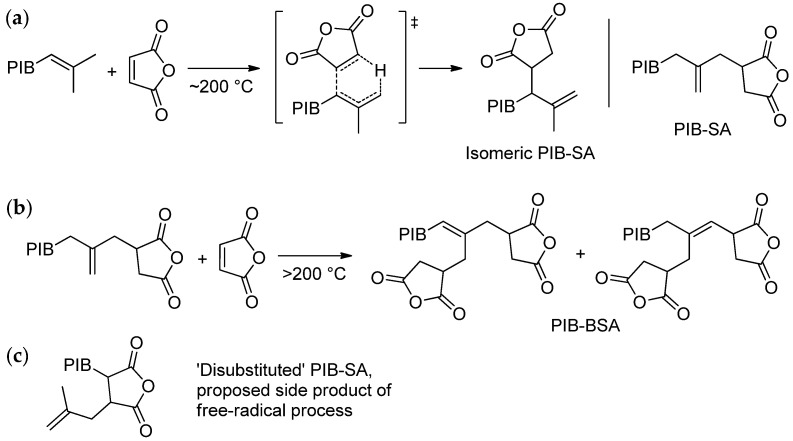
(**a**) Presumed reaction mechanism for the formation of the isomeric PIB-SA: the structure of conventional PIB-SA is presented for comparison; (**b**) reaction of PIB-SA with an excess of MA with a formation of PIB-BSA [131]; and (**c**) proposed structure of the side product of free-radical reaction [132].

**Figure 19:**
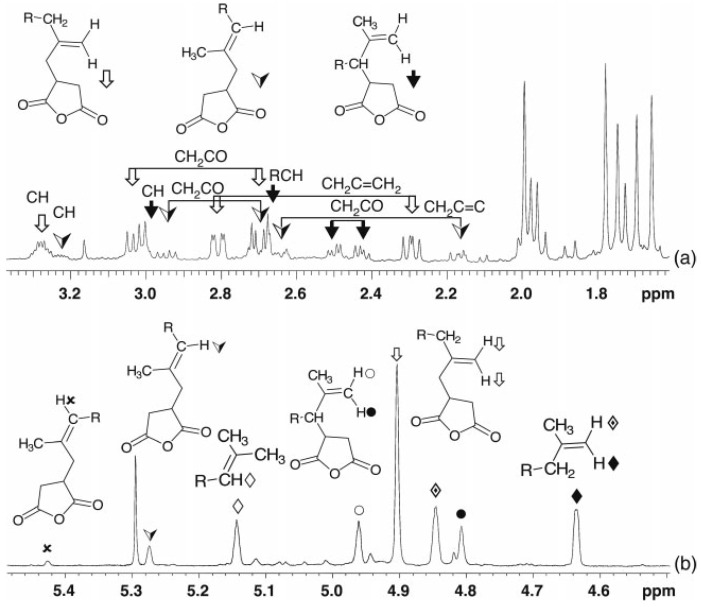
^1^H NMR (600 MHz, CDCl_3_, 25 °C) spectral region corresponding to (**a**) alkyl resonances of the end-groups of PIB-SA derivatives and (**b**) olefinic resonances of PIB-SA. Reprinted with permission from [131]. Copyright (2012) Wiley-VCH Verlag GmbH & Co.

The Alder-ene reaction between HRPIB and MA at ~200 °C proceeds mainly with the formation of a vinylidene adduct (Scheme 1a). However, detailed NMR studies have shown that other isomers of PIB-SA can also be identified in the reaction mixture [131] (Figure 19). A very important result reported in [131] was the identification of an *exo*-olefinic isomer of conventional PIB-SA, presumably formed by the reaction of MA with the PIB isomer containing tri-substituted olefinic end-group (Scheme 16a). Note that in other works, the formation of isomeric PIB-SA was attributed to the free-radical side reaction [132]. It is well known that at higher temperatures PIB-SA can react with an excess of MA with the formation of bis-succinic anhydride derivatives, PIB-BSA (Scheme 16b) [133]. It is quite likely that isomeric PIB-SA is able to react with the second molecule of MA. However, the products of the Alder-ene reaction of isomeric PIB-SA with MA were not detected during thorough analysis of the commercial sample of PIB-SA, whereas both isomers of ‘conventional’ PIB-BSA were detected [134].

^1^H NMR identification of PIB-BSA is a relatively challenging task because of the low content of PIB-BSA, the broad signal resulting from the slowly tumbling PIB-SA chains, and the presence of a forest of peaks in the region corresponding to the SA fragment. In a recent study of Frasca and Duhamel [135], PIB-SA samples were labeled with 1-pyrenemethylamine (180 °C, in *n*-decane). Detection of PIB-BSA was based on the preference of intramolecular aggregation of pyrene fragments that resulted in higher fluorescence decay for PIB-BSI-(pyrene)_2_ in comparison with PIB-BSI-pyrene. In further study of the products of the reaction of PIB-SA with different molar ratios of hexamethylene diamine [136], they found that PIBSA samples comprise 0.50, 0.38, and 0.12 molar fractions of PIB-SA, PIB and PIB-BSA, respectively.

**Figure 20:**
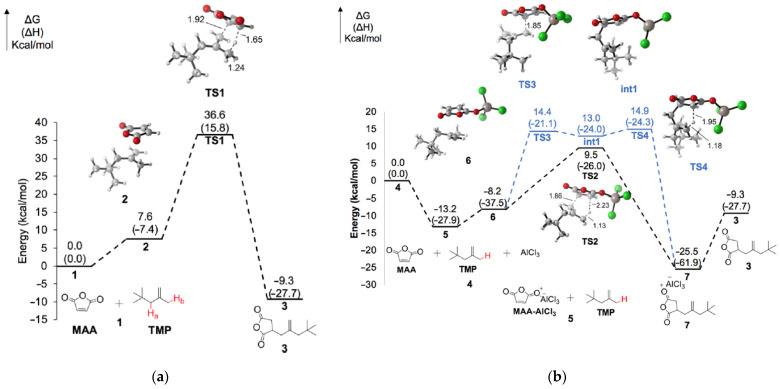
Reaction free energy (ΔG) profiles of the (**a**) concerted uncatalyzed and (**b**) AlCl_3_-catalyzed concerted (black) and stepwise (blue) mechanisms of the ene reaction between MA and Me_3_CCH_2_C(Me)=CH_2_. All energies are reported in kcal/mol. The values in parenthesis correspond to reaction enthalpies (ΔH). Reprinted with permission from [137]. Copyright (2021) American Chemical Society.

The results of DFT modeling of the mechanism of Alder-ene reaction between HRPIB and MA (M06-2X/6- 311+G(d,p) level of theory) were reported by Mpourmpakis et al. in 2021 [137]. First, they analyzed the conventional concerted mechanism of this reaction using Me_3_CCH_2_C(Me)=CH_2_ as a model of low-ME HRPIB molecule. Calculations showed that under uncatalyzed conditions, the ene reaction between MA and Me_3_CCH_2_C(Me)=CH_2_ proceeds by involving H_b_ (Figure 20a) with a Gibbs free activation energy (ΔG^≠^) of 36.6 kcal∙mol^−1^. The ene reaction involving H_a_ (not terminal) of Me_3_CCH_2_C(Me)=CH_2_ was also taken into consideration; the calculated ΔG^≠^ was 39.4 kcal∙mol^−1^. In our previous work [138], the calculated value of ΔG^≠^ for Alder-ene reaction of MA with 3-methyleneheptane was 34 kcal∙mol^−1^ (DFT, mpw1pw91/6-311g(d) level of theory), which corresponds to the results of Mpourmpakis et al.

In [137], an attempt of modeling a stepwise mechanism, involving a C−C bond formation TS, a zwitterionic intermediate, and an H-migration TS, was undertaken, but it failed. This modeling was successful for the systems containing Lewis acids. When samples of AlCl_3_ are used, it can be seen that a concerted mechanism is preferable (Figure 20b). Moreover, comparison of uncatalyzed and AlCl_3_-catalyzed ene reactions showed that the use of the AlCl_3_ catalyst lowers the Gibbs free activation energy (22.7 vs. 36.6 kcal∙mol^−1^). Similar results were obtained for TiCl_3_ and SnCl_2_, but with higher activation barriers. DFT modeling of the ene reaction was also conducted for InCl_3_, GaCl_3_, SnCl_4_, and TiCl_4_. Among the different Lewis acids, the calculations revealed AlCl_3_ and InCl_3_ to be the most active catalysts.

In a recent study on the kinetics of the reaction of HRPIB with MA [132], the composition of the reaction mixture was studied in a temperature interval of 150–180 °C. Experimental value of the ΔG^≠^ for the thermal ene reaction (34–35 kcal∙mol^−1^) was in good agreement with the results of DFT modeling (36.6 kcal∙mol^−1^, [137]). However, conversion of HRPIB and MA in the presence of 3–8 mol% AlCl_3_ was essentially unaffected compared to the background reaction, but the proportion of *endo*-PIB increased compared to the uncatalyzed conditions as AlCl_3_ loading increased. Given the clear calculated drivers for Lewis acid acceleration and the fact that this is a successful strategy in other Alder-ene reactions [139], the authors of [132] proposed that the minor by-products formed during the reaction between MA and HRPIB are also strong sequestering agents for AlCl_3_. More recently, Mpourmpakis et al. proposed the use of EtAlCl_2_ instead of AlCl_3_, which showed that alkylaluminum chloride retained relatively low activation barriers like AlCl_3_ but formed less stable complexes with PIB-SA [140].

From a practical point of view, the use of excess MA in an ene reaction with HRPIB, that results in the formation of ‘SA-excessive’ adducts like PIB-BSA, entails substantial improvement in the characteristics of the corresponding PIB-SI [141,142]. Similar observations prompted the chemists of the Lubrizol Corp. to find alternative synthetic approaches to PIB-BSA analogs. In [143], they suggested to conduct the reaction between HRPIB and MA in the presence of free-radical initiators. For example, (*^t^*BuO)_2_-initiated interaction of HRPIB with MA was carried out at 170 °C within 2 h, obtaining a colorless product with a higher acid number in comparison with the product of thermal ene reaction. The molecular structure of the products of this free-radical process remains unexplored. In [144], PIB-SA was synthesized from the commercial HRPIB (*M*_n_ = 4.17 kDa and *Đ*_M_ = 1.98) and MA with the addition of benzoyl peroxide. The reaction was conducted at 150–190 °C for 24 h; however, the mechanism of the reaction was not discussed, and the reported structure of the product corresponded to the thermal ene reaction mechanism.

PIB-SA exhibits an amphiphilic molecule with unusual conformational behavior. In 2022, Johnson et al. reported the results of a theoretical study on PIB-SA containing up to nine repeat units [145]. They used a combination of classical potential calculations for generating conformations and DFT (B3LYP/def2-SVP level of theory) for optimizing the structures and computing the dipole moments. The computed average dipole moments decreased from 4.8 ± 0.4 to 3.9 ± 0.6 D with the increase in chain lengths.

#### 6.1.2. PIB-SI and Related Compounds

The reaction of PIB-SA with diamines (Scheme 1d) is the basis for the well-operating, large-scale production of ashless dispersants and as such is not of interest in our review. However, a number of works that are devoted to the determination of the molecular structure and spectral characteristics of PIB-SI have been published recently. In particular, as was shown in [144,146], the quantitative NMR study of diethylenetriamine, triethylenetetramine, tetraethylenepentamine, and pentaethylenehexamine-based PIB-SIs is hindered by hydrogen bonding between the polyamine linker and the succinimide carbonyls; this problem remains critical for the post-modified products of PIB-SI after the reaction with ethylene carbonate [147]. An alternative method of the analysis of PIB-SI was proposed, which was based on fluorescent quenching, and it was found that the fluorescence of the succinimide groups decreased with the increasing number of secondary amine fragments in the polyamine linker [146], whereas the quenching of succinimide moieties by urethane groups was much less efficient [147].

**Scheme 17:**
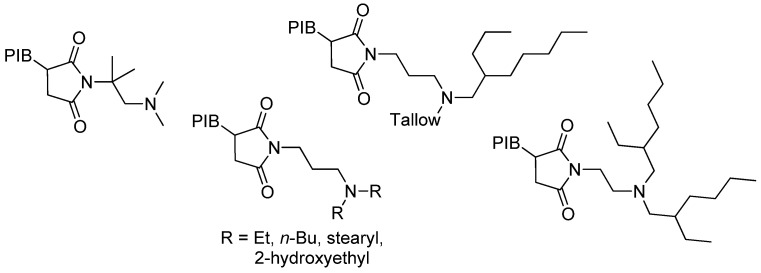
Molecular structures of prospective ashless dispersants [10].

However, the search for prospective PIB-SA-based additives has continued. In particular, the chemists of the Lubrizol Corp. synthesized a series of PIB-SA-based derivatives of sterically hindered diamines (Scheme 17) to reduce deposits and corrosion on the internal parts of engines [10]. The chemists of Innospec, using an example of Me_2_N(CH_2_)_3_NH_2_-derived PIB-SI, have demonstrated the higher efficiency of additives prepared from succinic anhydrides with a higher PIB-BSA content [141,142].

In [148], Ponzoni et al. analyzed a series of virtual PIB-SI molecules derived from known reference substrates, and developed a comprehensive model for the search of prospective ashless dispersants using machine learning techniques. In the future, the practical significance of this work will be demonstrated.

The study on the colloidal behavior of carbon black (average particle size 126 ± 3.5 nm) in the presence of commercial diamine-based bis(PIB-SI) dispersant in *n*-hexane and *n*-decane [149] showed that the magnitude of effective surface charge of carbon nanoparticles, determined using electrophoretic mobility measurements, decreases with an increase in PIB-SI concentration. Dynamic light scattering (DLS) and UV spectral studies showed that minimal aggregation of the carbon nanoparticles in *n*-decane is observed at a PIB-SI concentration of 5 wt%, which correlates with the optimized formulations for Group III and IV engine oils. An amphiphilic nature of PIB-SI was used in surface-capping of MoO_3_; the nanoparticles modified with zinc dialkyldithiophosphate [150] demonstrated a synergistic tribological effect, reduced friction and caused wear in the diamond-like carbon coating under boundary lubrication.

### 6.2. Recent Studies on Other Methods of HRPIB Functionalization

Acid-catalyzed reaction of HRPIB with nitriles, resulting in amide formation (Ritter reaction), was conducted successfully, but only in the case of low-MW oligomers (C_12_–C_20_). In the case of commercial HRPIBs, products of acid-catalyzed isomerization were detected [151].

**Scheme 18:**
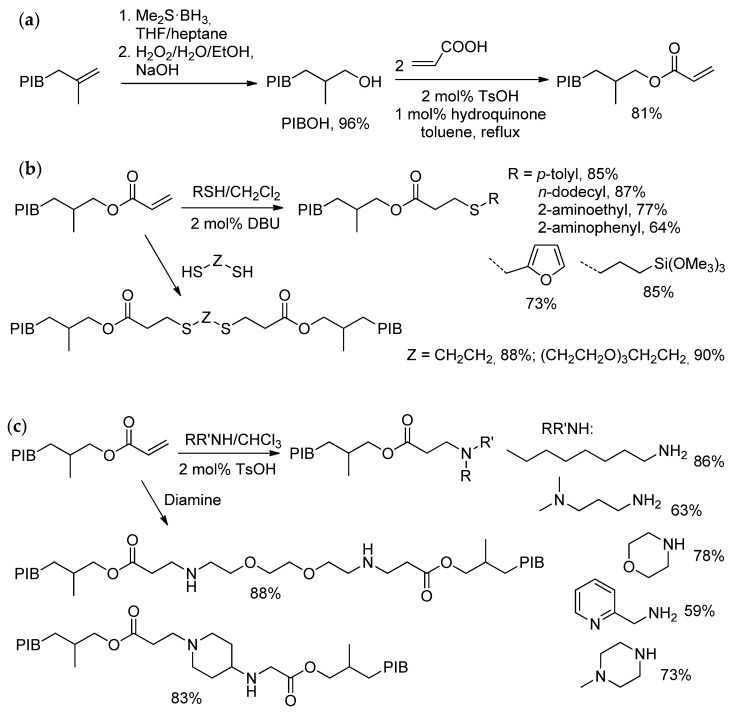
(**a**) Synthesis of PIB*_exo_*OH and PIB acrylate; (**b**) thiol-ene reactions of PIB acrylate; and (**c**) Aza–Michael reactions of PIB acrylate [152] (DBU—1,8-diazabicyclo [5.4.0]undec-7-ene; TsOH—*p*-toluenesulfonic acid).

Based on commercial HRPIB (*M*_n_ = 1.03 kDa and *Đ*_M_ = 1.55) Kulai et al. [152] synthesized corresponding PIB alcohol (PIB*_exo_*OH) via hydroboration and subsequent oxidation. Acylation of PIB*_exo_*OH with acrylic acid in the presence of *p*-toluenesulfonic acid (TsOH) afforded a PIB acrylate with an 81% yield (Scheme 18a). This PIB acrylate was studied in thiol-ene addition (Scheme 18b) and aza-Michael reaction (Scheme 18c).

**Scheme 19:**
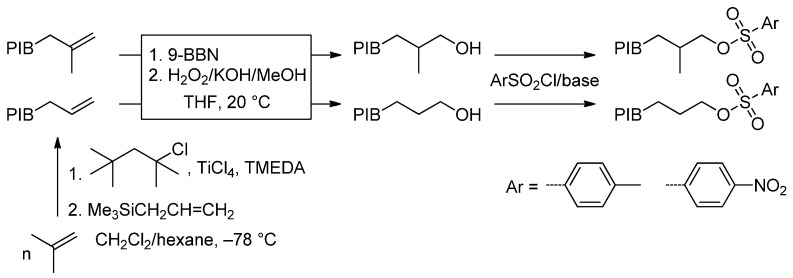
Synthesis of arenesulfonyl-ended PIBs derived from *exo*-olefin-ended and allyl-terminated polymers [153].

PIB*_exo_*OH was obtained in a similar way, using 9-borabicyclo[3.3.1]nonane (9-BBN) as a hydroboration reagent [153]. In addition, termination of quasiliving carbocationic polymerization of IB (*^t^*BuCH_2_CMe_2_Cl/TiCl_4_/N,N,N′,N′-tetramethylethylenediamine) by Me_3_SiCH_2_CH=CH_2_, followed by hydroboration/oxidation afforded PIB*_all_*OH. PIB*_exo_*OH and PIB*_all_*OH was further converted into PIB-OSO_2_Ar (Ar = *p*-tolyl and 4-nitrophenyl, respectively) (Scheme 19) [153]. A significant difference in the reactivity was found, i.e., PIB*_all_*OH reacted faster than PIB*_exo_*OH, presumably because of the steric reasons. While quantitative tosylation was achieved with PIB*_exo_*OH, nosylation led to PIB*_exo_*ONs with a functionality of ~90%. It was also found that PIB*_all_*OTs react further with the chloride anion formed during tosylation, leading to the yield of a chlorine-ended PIB (PIB*_all_*Cl). The latter reaction illustrates high synthetic potential of PIB sulfonates.

**Scheme 20:**
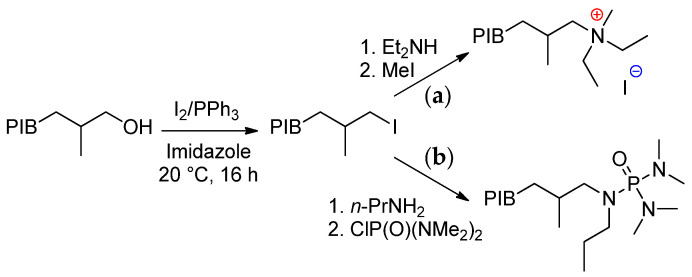
Synthesis of (**a**) PIB-bound quaternary ammonium salt [154] and (**b**) PIB-bound phosphoramide [155].

Due to high lipophilicity, functionalized PIBs are prospective candidates for use in the two-phase processes. Transformation of PIB*_exo_*OH (*M*_n_ = 1 kDa) to corresponding iodides (I_2_/PPh_3_, imidazole in CH_2_Cl_2_), followed by the treatment with Et_2_NH and quaternization via MeI afforded PIB-ammonium salts (Scheme 20a) that have demonstrated high efficiency as phase-transfer agents for S_N_2 reactions [154]. In 2020 [155], Fu and Bergbreiter described the synthesis of a PIB-bound hexamethylphosphoramide (HMPA) analog starting from PIB*_exo_*OH (Scheme 20b), and its applications as a recyclable catalyst in allylation of aldehydes (ArCHO + Cl_3_SiCH_2_CH=CH_2_) and reduction in enones with SiHCl_3_ in a poly-α-olefin (PAO) solvent (hydrogenated dec-1-ene trimer was used as a PAO). These studies have shown that PIB-bound phosphoramide/PAO is a competent alternative to HMPA in both the processes as a recyclable catalyst.

Interaction of PIB*_exo_*I, obtained from HRPIB (*M*_n_ = 450 Da) with different N-nucleophiles (secondary amines, imidazole, and substituted imidazoles), led to the products of nucleophilic substitution [156]. These functionalized PIBs in combination with PAO (hydrogenated dec-1-ene trimer) were studied in the extraction of phenols from water. For example, using an imidazole-functionalized PIB at a concentration of 0.1 M in PAO, >99% of the bisphenol A (BPA) was extracted from the aqueous solution with a concentration of 200 mg∙L^−1^. Other chlorinated, brominated, and alkylated phenols (200–500 mg∙L^−1^) were also extracted with >95% efficiency. Recycling of the PB-imidazole/PAO phase containing sequestered phenol was easily performed by subsequent mixing with solid NaOH. These results are of great importance due to the high toxicity of BPA, which is widely used in the production of polycarbonates [157,158].

**Scheme 21:**
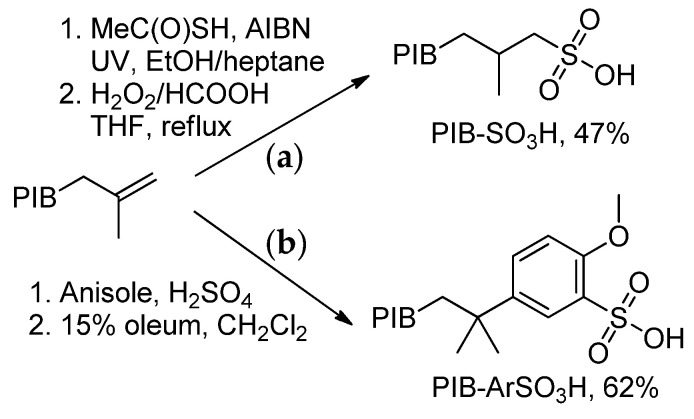
Synthesis of PIB-bound (**a**) alkane and (**b**) arenesulfonic acids [159].

In 2021, Bergbreiter et al. proposed the use of fully recyclable PAO and soluble PAO-anchored PIB-bound sulfonic acid catalysts [159]. HRPIB with *M*_n_ = 432 was subjected to a thiol-ene reaction followed by oxidation affording PIB-SO_3_H with a 47% yield (Scheme 21). PIB analog of arenesulfonic acids, PIB-ArSO_3_H, was synthesized in two stages in a 62% yield via alkylation of anisole, followed by sulfonation (Scheme 21). Catalytic potential of sulfonic acids was studied during the reaction of PhCH_2_OH with 3,4-dihydro-2*H*-pyran (5 mol% of the catalysts and >90% yields over 10 cycles); comparison showed that the *p*-dodecylbenzenesulfonic acid/PAO system lost activity after the third cycle. Catalytic potentials of PIB-SO_3_H and PIB-ArSO_3_H were also estimated for esterification and three-component condensation between PhCOH, urea and acetoacetic ester; at least 80% yields of the products were achieved after recycling seven times and more [159].

**Scheme 22:**
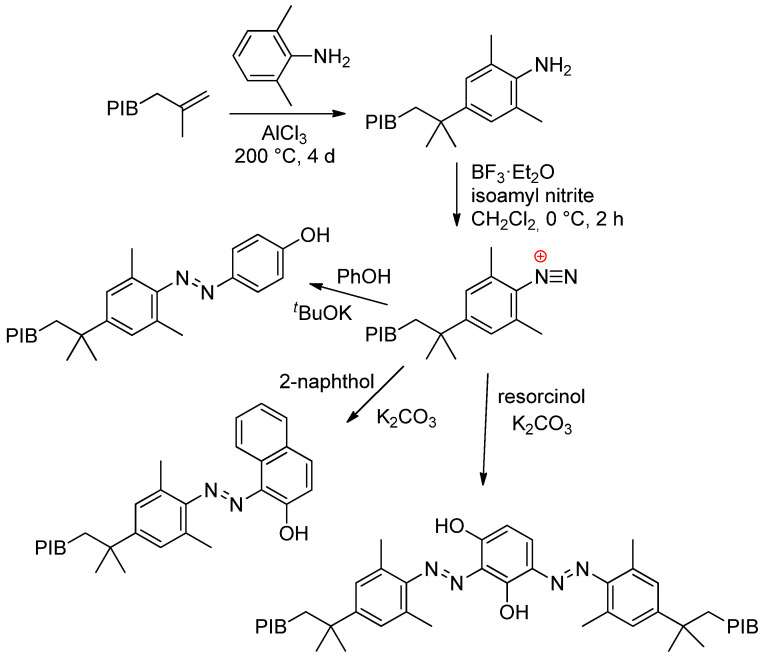
Synthesis of PIB-bound azo dyes [160].

It is worth pointing out here that alkylation of the active aromatic compounds (substituted anilines) by HRPIB (*M*_n_ = 1 kDa) was efficiently used in the synthesis of lipophilic azo dyes [160] (Scheme 22), that are the colored probes used in the study of high-boiling non-toxic solvents, including PAOs. Alkylation of 6-phenoxyhexan-1-amine by PIB-Cl resulted in an amino-functionalized PIB that was used for the stabilization of nanocrystalline semiconductors (CdSe and PbS) [161].

**Scheme 23:**
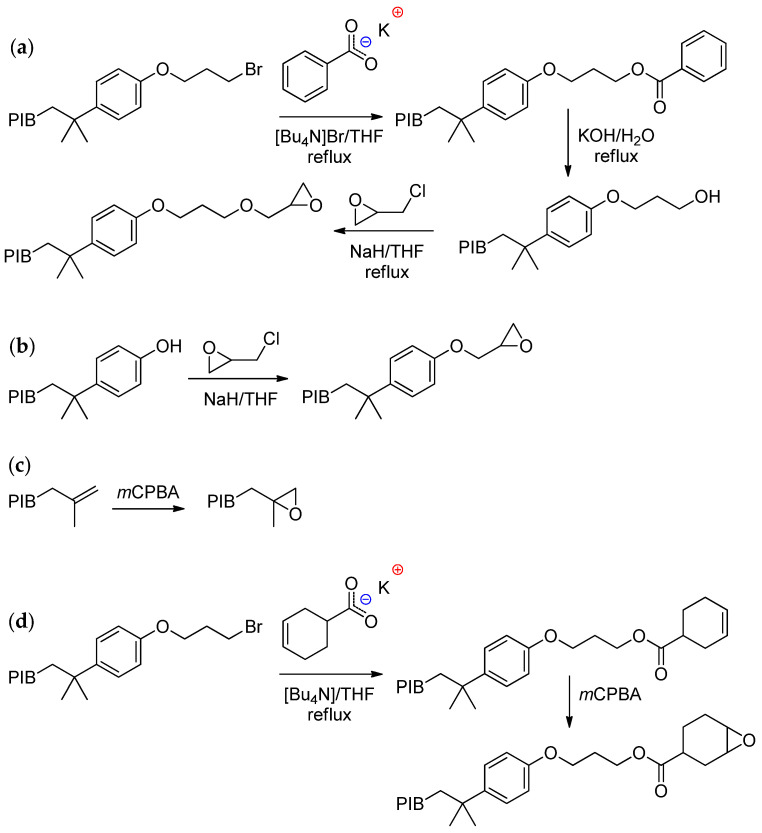
Synthesis of PIB telechelic prepolymers with various epoxide functionalities: (**a**) PIB aliphatic glycidyl ether; (**b**) PIB phenyl glycidyl ether; (**c**) PIB exo-olefin epoxide; and (**d**) PIB cyclohexene epoxide [163] (*m*CPBA—3-chloroperbenzoic acid).

PIB-substituted phenols were synthesized using alkylation of phenol by HRPIBs, and catalyzed by PhOH∙BF_3_ in *n*-hexane/toluene media [162]. The structure of the reaction products is not entirely clear; ^1^H NMR data, presented in this patent, raise questions about the assignment of the signals. On the other hand, in [163], the structure of the alkylation product was clearly proven. In this work, bifunctional HRPIB and the product of its reaction with phenol were used as starting compounds for the synthesis of PIBs with oxirane functionality; the reactions on one chain-end fragment are presented in Scheme 23. Alkylation of phenol by three-arm star HRPIB (prepared using 1,3,5-C_6_H_3_(CMe_2_Cl)_3_ initiator and FeCl_4_/^i^PrOH catalysts), followed by acylation with methacryloyl chloride, resulted in a highly reactive photo-crosslincable polymer that was used for matrix stabilization of quantum dots [164].

**Scheme 24:**
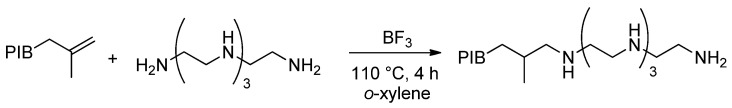
BF_3_-catalyzed reaction of HRPIB with pentaethylenehexamine [165].

In 2021 [165], Pebriani et al. published a controversial report on the synthesis of diamine derivatives of PIB based on direct acid-catalyzed interaction between HRPIB and pentaethylenehexamine. The structure of the reaction product (Scheme 24), proposed by the authors, was confirmed via FT-IR spectra. In the absence of NMR data, this structure (as a result of nucleophilic addition to =CH_2_) raises reasonable doubts from a mechanistic point of view. Note that in another study [166], this process was called ‘electrophilic substitution’ (no NMR data). Some doubt is also expressed regarding the choice of the solvent (*o*-xylene) that can react with HRPIB.

### 6.3. PIB-Based Copolymers

High reactivity of *exo*-olefin- and –CMe_2_Cl-terminated PIBs have been successfully used for the synthesis of amphiphilic block copolymers with excellent prospects for biomedical application and materials’ design [167]. In this section, we provide some practical examples of the preparation and applications of PIB copolymers.

Highly reactive ‘Cl-allyl-PIB-allyl-Cl’ copolymers (see Scheme 7) were hydrolyzed to the corresponding diols using aqueous tetrabutylammonium hydroxide in THF (reflux, 22 h, and 98.2% conversion); these diols in a mixture with butane-1,4-diol were further used for polyuerthane preparation through the reaction with 4,4′-methylenebis(phenyl isocyanate) in the presence of Sn(Oct)_2_ [68]. In subsequent studies [168], the synthesis of a copolymer was optimized to achieve mechanical characteristics and biocompatibility suitable for a fully synthetic heart valve. With the use of freshly distilled 4,4′-methylenebis(phenyl isocyanate) and increasing the PIB diol concentration in the reaction mixture, a copolymer with an increased MW (>100 kDa), a tensile strength of ≈32 MPa, an elongation of ≈630%, and a toughness of >4.0 J was achieved. This material had an impressive fatigue life, >1 million cycles around 1 MPa SED and >0.5 million at 2–3 MPa SED; it exhibited no calcification, and had less protein adsorption and cell attachment in comparison with silicone–rubber materials. A similar copolymer, synthesized from (4-HOC_6_H_4_)-PIB-(4-C_6_H_4_OH), was proposed for medical applications in a recent patent of Medtronics Inc. [169].

Ring-opening polymerization-induced self-assembly (ROPISA) was achieved by employing PIB-OH, diphenyl phosphate, cyclohexane, and ε-caprolactone (ε-CL) and/or δ-valerolactone (δ-VL). ROP at 80 °C interfered with the crystallization and in situ fixation, which readily reorganized nanoparticles and formed spherical micelles or precipitate. However, at 20 °C, fibrillar nanoparticles were gradually formed and stabilized according to the classical polymerization-induced self-assembly (PISA) mechanism [170].

PIB-OH was also used as a starting compound for the synthesis of chain transfer agent PIB-OC(O)CH_2_CH_2_C(Me)(CN)SC(S)SC_12_H_25_ for reversible addition-fragmentation chain transfer (RAFT) polymerization of fatty ester methacrylates [171]. The block copolymers showed varied crystalline behavior depending on the chain length of the fatty acids. PIB-Br-initiated alternating RAFT polymerization was also used in the synthesis of the copolymer of *tert*-butoxy carbonyl (Boc)-leucine containing a maleimide unit and Boc-alanine containing a styrene unit. Its deprotection resulted in an amphiphilic copolymer with free –NH_3_^+^ and –COO^−^ functionalities in an alternating fashion that demonstrated pH-responsive self-assembly [172]. A similar approach was used in the synthesis of PIB block copolymers containing aninonic (acrylic acid monomer), neutral (MeO(CH_2_CH_2_O)_4_-actylate monomer) and cationic ((Boc)-leucine-OCH_2_CH_2_O-acrylate monomer) fragments, designed for ion transport through the liposomal membrane [173]. RAFT copolymerization was also used for the synthesis of PIB-containing copolymer with glucose-decorated hydrophilic segment that showed high efficiency in the protection of the insulin aggregation [174].

The prospects of using PIB blocks in the design of stretchable semiconducting polymers was demonstrated on the example of ABA-type architecture comprising PIB and poly(naphthalenediimide–bithiophene) with various compositions [175].

Commercial PIB-SA was introduced to polycondensation with glycerol oligomers with a formation of 1:1 adducts and relatively high-MW polymers [176]. The optimal molar ratio of PIB-SA to polyglycerol was found to be 1:1.

Commercial HRPIB derivatives of the formula (PIB)CH_2_CH(Me)CH_2_CH_2_NH_2_ [177] were acylated with methacrlyc anhydride; the resulting amides were copolymerized with (meth)acrylates (*tert*-butyl-peroxyethylhexanoate as an initiator, *tert*-dodecyl mercaptan as a transfer agent, at 95 °C, and with isoparaffin as a solvent) [178]. Similar methacrylic monomers were prepared via acylation of PIB-substituted phenols [162,179]. Resulting comb copolymers were proposed as viscosity index improvers for engine oil formulations.

## 7. Conclusions

The development of new, cost-efficient and environmentally friendly catalysts for replacing commercially used BF_3_ in the synthesis of HRPIBs is still relevant. Since 2010, a number of alternative catalytic systems have been studied. The most prospective initiators of the IB polymerization for the production of HRPIBs are the complexes of metal chlorides with ethers, the complexes of alkylmetal chlorides with ethers, and heterogeneous systems mainly based on the metal chloride/ether adducts—the first-_,_ second-, and third-generation catalysts—according to the classification proposed by S. Kostjuk in 2021 [2].

Among the metal chloride/R_2_O catalytic systems, AlCl_3_/R_2_O could be a good alternative to BF_3_-based catalysts; however, the problem of low activity and poor solubility of the catalytic complexes in aliphatic media should be solved. Further improvement in similar catalysts in terms of the selectivity of β-H elimination (preferably in *n*-hexane or in C4 feed), via both the design of new Lewis acids and new electron donors, can be complicated by the formation of relatively high-MW PIBs when highly active catalyst could be found [22]. Also note that aromatic solvents are still proposed to be used in IB polymerization [80,81,84,126], even though aromatic hydrocarbons can undergo alkylation [47] and depolymerize PIBs in the presence of acids [51].

Using C4 hydrocarbon feeds instead of pure IB is a growing trend in modern technologies of HRPIB manufacturing [55,56]. Therefore, new-generation catalysts should be active in a non-polar reaction medium and inert towards but-1-ene and but-2-enes, that hardly fits with a high catalytic activity in cationic processes. The first results in this field have already been published [34,78,84,89,97], but further studies are required on the use of hydrocarbon mixtures with comprehensive analyses of the microstructure of polymers.

In the development of heterogeneous catalysts for the production of HRPIBs, certain analogies with the development of supported α-olefin polymerization catalysts arise. Except for the problem of the resulting polymer particle morphology (irrelevant for more soluble PIBs), the problems associated with the efficient and stable supporting of the active species, maximizing the surface area, and selecting the donor additives seem common for both processes. These problems have been solved successfully for Ziegler–Natta catalysts, and we can also expect this progress in the area of heterogeneous catalysis for IB cationic polymerization in the near future.

Finally, one cannot fail to note the structural similarity of HRPIBs and methylenealkanes, including vinylidene dimers and oligomers of α-olefins [180,181]. Apparently, the methods of HRPIB functionalization can be successfully applied in methylenealkane chemistry, and vice versa, especially in view of the progress achieved recently in catalytic transformations of methylenealkanes [182,183,184,185,186,187].

## Figures and Tables

**Table 1:** Ionization of [PIB-Cl] = 0.015 M with [Et_2_AlCl] = 0.015 M in hydrocarbons for 30 min in the presence of [DTBP] = 0.015 M [31].

T, °C	Olefin End-Group Distribution, %			
	tri-Substituted	*exo*-	*endo*-	tetra-Substituted
−40	77	8	4	11
0	61	18	9	12
20	0	40	20	40

**Table 2:** Polymerization of IB using AlCl_3_/R_2_O catalysts at 0 °C. [IB] = 4 M in hexane; reaction time 1 h [80,81].

Entry	[AlCl_3_], M	[^i^Pr_2_O], M	[Bu_2_O], M	Conv., % ^1^	*M*_n_, kDa ^2^	*exo*-, %
1	0.01	0.01	–	98	1.3	56
2	0.01	–	0.01	28	2.8	77
3	0.01	0.005	0.005	98	1.5	78
4	0.01	0.0025	0.0075	97	1.5	66
5	0.01	0.0075	0.0025	62	2.1	79
6	0.01	0.001	0.009	30	2.8	76
7	0.01	0.009	0.001	98	1.4	58
8	0.01	0.006	0.006	98	1.0	84
9	0.01	0.0075	0.0075	72	0.8	85
10	0.01	0.01	0.01	14	3.2	85
11	0.005	0.0025	0.0025	74	2.6	77
12 ^3^	0.01	0.005	0.005	98	1.4	77
13 ^4^	0.01	0.005	0.005	95	1.5	79

^1^ Determined gravimetrically based on IB. ^2^ Determined using ^1^H NMR. ^3^ Toluene was used as a solvent. ^4^ THF was used instead of Bu_2_O.

**Table 3:** Polymerization of IB using AlCl_3_ and/or EtAlCl_2_/R_2_O catalysts at 0 °C. [IB] = 4 M in hexane; reaction time 1 h [80,81].

Entry	[AlCl_3_], M	[EtAlCl_2_], M	[^i^Pr_2_O], M	[Bu_2_O], M	Conv., % ^1^	*M*_n_, kDa ^2^	*exo*-, %
1	0.01	–	0.01	–	98	1.3	56
2	–	0.01	0.01	–	7	4.2	82
3	0.005	0.005	0.01	–	98	1.4	84
4	0.0025	0.0075	0.01	–	85	1.8	80
5	0.0075	0.0025	0.01	–	98	1.4	64
6	0.001	0.009	0.01	–	10	3.8	83
7	0.009	0.001	0.01	–	98	1.3	55
8	0.01	0.01	0.02	–	98	1.0	81
9	0.0025	0.0025	0.005	–	70	2.8	80
10	0.01	–	–	0.01	28	2.8	77
11	–	0.01	–	0.01	<5	4.2	88
12	0.005	0.005	–	0.01	20	3.4	82
13 ^3^	0.005	0.005	0.01	–	98	1.3	83

^1^ Determined gravimetrically based on IB. ^2^ Determined using ^1^H NMR. ^3^ Xylene was used as a solvent.

**Table 4:** Cationic polymerization of IB in the presence of alkoxy aluminum chlorides as co-initiators in *n*-hexane at 10 °C [106] ^1^.

Entry	Init.	Conv., %	*M*_n_^SEC^, kDa	*M*_n_^NMR^, kDa	*Ð*_M_	End Group Distribution (mol%)			
						*exo*-	*endo*- + tri-	tetra-	PIB-Cl
1	HexOAlCl_2_	93	8.20	6.96	2.9	58	18	22	2
2	BuOAlCl_2_	100	5.62	5.89	3.7	46	26	23	5
3	^i^PrOAlCl2	99	4.27	3.71	5.4	32	33	35	0
4	PhOAlCl_2_	84	0.50	0.43	9.4	14	37	48	1
5	(BuO)_0.8_AlCl_2.2_	100	0.81	0.46	21.3	0	66	34	0
6	(BuO)_1.2_AlCl_1.8_	44	18.10	15.20	2.0	71	16	12	1
7 ^2^	(BuO)_1.2_AlCl_1.8_	81	12.44	13.20	2.6	83	14	0	3

^1^ Polymerization conditions: [co-initiator] = 38 mM′, [IB] = 5.8 M′, reaction time 10 min. ^2^ Reaction time 30 min.

**Table 5:** Polymerization of IB in *n*-hexane for 30 min catalyzed by the emim-Cl/AlCl_3_ (χ(AlCl_3_) = 0.6) ionic liquid (IL) in the presence of ethers [108] ^1^.

Entry	T, °C	Ether	Ether/IL, mol	Conv., %	*M*_n_^SEC^, kDa	*M*_n_^NMR^, kDa	*Ð*_M_	End-Group Distribution (mol%)			
								*exo*-	*endo*- + tri-	tetra-	PIB-Cl
1	0	–	–	100	1.50	1.36	6.8	2	74	24	0
2	0	^i^Pr_2_O	0.5	51	3.94	3.34	2.0	92	4	3	1
3	0	Bu_2_O	0.5	12	9.35	5.17	1.6	86	6	5	3
4	0	^t^BuOMe	0.5	90	2.70	1.51	4.0	3	69	26	2
5 ^2^	0	^i^Pr_2_O	1.0	28	4.90	3.24	1.9	95	2	1	2
6	0	^i^Pr_2_O	0.4	57	7.75	4.46	1.7	84	6	8	2
7 ^3^	0	^i^Pr_2_O	0.35	62	6.50	4.51	1.9	89	4	4	3
8	0	^i^Pr_2_O	0.3	71	12.90	9.57	1.8	47	30	19	4
9	−20	^i^Pr_2_O	0.5	54	9.20	4.63	3.3	36	45	17	2
10	−10	^i^Pr_2_O	0.5	13	5.76	6.52	2.9	67	15	15	3
11	10	^i^Pr_2_O	0.5	59	5.60	4.65	2.0	90	6	3	1
12 ^4^	10	^i^Pr_2_O	1.0	57	3.47	2.90	2.7	69	11	17	3
13 ^5,6^	10	^i^Pr_2_O	0.9	55	1.40	1.06	3.6	84	5	7	<1

^1^ [emim-Cl–AlCl_3_] = 22 mM; [IB] = 5.2 M; [R_2_O] = 11 mM. ^2^ [emim-Cl–AlCl_3_] = 11 mM. ^3^ [emim-Cl–AlCl_3_] = 33 mM. ^4^ AlCl_3_·^i^Pr_2_O (22 mM) was used as a catalyst instead of an IL. ^5^ EtAlCl_2_∙ ^i^Pr_2_O was used as a catalyst. ^6^ In addition to *exo*-, *endo*-, tri- and tetra-substituted olefinic groups, ~4% of coupled polymer chains are presented.

**Table 6:** Cationic polymerization of IB with the binary systems phenols/Al(C_6_F_5_)_3_ in toluene at −20 °C. [125] ^1^.

Entry	Phenol	Yield, %	*M*_n_^SEC^, ^2^ kDa	*M*_n_^NMR^, ^3^ kDa	*Ð*_M_	End Group Distribution (mol%)			
						*exo*-	*endo*- + tri-	tetra-	Coupled
1	1a	12.7	469	269	1.59	65.4	32.0	1.5	1.1
2	1b	59.5	126	205	1.63	61.4	31.0	5.5	2.1
3	1c	67.5	98	166	1.70	59.5	13.2	25.9	1.4
4	1d	93.0	72	169	2.37	63.1	28.5	7.1	1.3
5	2a	76.2	16	49	3.06	46.3	28.5	18.4	6.8
6	2b	95.5	45	114	2.52	54.6	35.9	8.3	1.2
7	2c	14.8	93	175	1.87	61.1	36.9	0.6	1.4
8	2d	34.4	119	192	1.61	56.2	36.4	6.7	0.7

^1^ Polymerization conditions: [IB]:[phenol]:[Al] = 3500:1:1, [Al(C_6_F_5_)_3_] = 10 μmol, reaction time 1 h. ^2^ Determined via SEC in THF at 40 °C against polystyrene standard. ^3^ Determined using ^1^H NMR.

**Table 7:** Isomerization of HRPIB Glissopal 1000 in *n*-hexane at 80 °C. [130] ^1^.

Reaction Time, h	A, C6, C8 ^2^	B ^2^	C1, C2, B, C7 ^2^	C3, C4, C5 ^2^	*M*_n_, kDa
0	87.1	8.7	11.4	1.5	1030
1	38.8	45.0	56.4	6.6	1100
2	33.3	44.8	56.8	9.9	1110
3	31.1	42.1	56.2	12.7	1120
4	28.7	40.1	54.7	16.6	1150
5	26.8	37.8	52.6	20.6	1080
6	25.0	34.6	51.0	24.0	1050

^1^ Reaction conditions: HRPIB (40 g), *n*-hexane (40 g), Al_2_O_3_ (33 g), molecular sieves 5Å (33 g). ^2^ See Scheme 15.

## Data Availability

Not applicable.
